# Solution-Processable Indenofluorenes on Polymer Brush Interlayer: Remarkable N-Channel Field-Effect Transistor Characteristics under Ambient Conditions

**DOI:** 10.1021/acsami.3c07365

**Published:** 2023-08-15

**Authors:** Ayse Can, Ibrahim Deneme, Gokhan Demirel, Hakan Usta

**Affiliations:** † Department of Nanotechnology Engineering, Abdullah Gül University, 38080 Kayseri, Turkey; ‡ Bio-inspired Materials Research Laboratory (BIMREL), Department of Chemistry, Gazi University, 06500 Ankara, Turkey

**Keywords:** n-type semiconductor, low LUMO materials, organic field-effect transistor, alkyl chain engineering, thin-film crystallinity

## Abstract

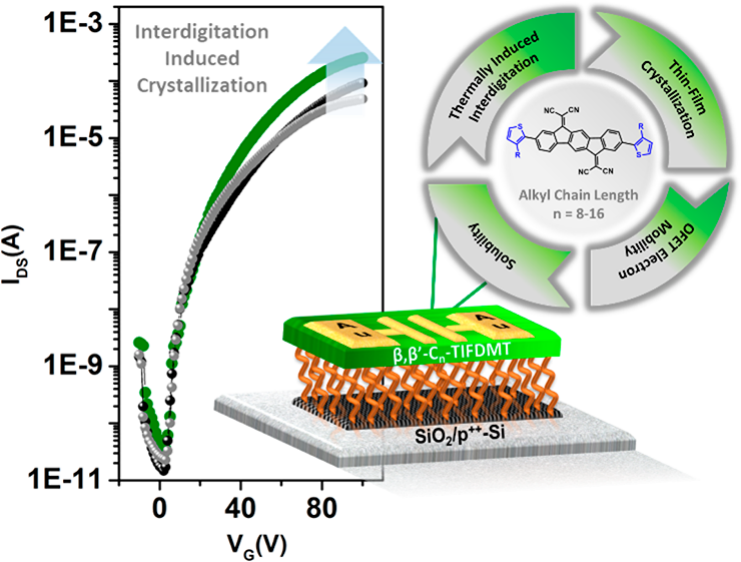

The development of solution-processable n-type molecular semiconductors that exhibit high electron mobility (μ_e_ ≥ 0.5 cm^2^/(V·s)) under ambient conditions, along with high current modulation (*I*_on_/*I*_off_ ≥ 10^6^–10^7^) and near-zero turn on voltage (*V*_on_) characteristics, has lagged behind that of other semiconductors in organic field-effect transistors (OFETs). Here, we report the design, synthesis, physicochemical and optoelectronic characterizations, and OFET performances of a library of solution-processable, low-LUMO (−4.20 eV) 2,2′-(2,8-bis(3-alkylthiophen-2-yl)indeno[1,2-*b*]fluorene-6,12-diylidene)dimalononitrile small molecules, **β,β′-C**_***n***_**-TIFDMTs**, having varied alkyl chain lengths (*n* = 8, 12, 16). An intriguing correlation is identified between the solid–isotropic liquid transition enthalpies and the solubilities, indicating that cohesive energetics, which are tuned by alkyl chains, play a pivotal role in determining solubility. The semiconductors were spin-coated under ambient conditions on densely packed (grafting densities of 0.19–0.45 chains/nm^2^) ultrathin (∼3.6–6.6 nm) polystyrene-brush surfaces. It is demonstrated that, on this polymer interlayer, thermally induced dispersive interactions occurring over a large number of methylene units between flexible alkyl chains (i.e., zipper effect) are critical to achieve a favorable thin-film crystallization with a proper microstructure and morphology for efficient charge transport. While C_8_ and C_16_ chains show a minimal zipper effect upon thermal annealing, C_12_ chains undergo an extended interdigitation involving ∼6 methylene units. This results in the formation of large crystallites having lamellar stacking ((100) coherence length ∼30 nm) in the out-of-plane direction and highly favorable in-plane π-interactions in a slipped-stacked arrangement. Uninterrupted microstructural integrity (i.e., no face-on (010)-oriented crystallites) was found to be critical to achieving high mobilities. The excellent crystallinity of the C_12_-substituted semiconductor thin film was also evident in the observed crystal lattice vibrations (phonons) at 58 cm^–1^ in low-frequency Raman scattering. Two-dimensional micrometer-sized (∼1–3 μm), sharp-edged plate-like grains lying parallel with the substrate plane were observed. OFETs fabricated by the current small molecules showed excellent n-channel behavior in ambient with μ_e_ values reaching ∼0.9 cm^2^/(V·s), *I*_on_/*I*_off_ ∼ 10^7^–10^8^, and *V*_on_ ≈ 0 V. Our study not only demonstrates one of the highest performing n-channel OFET devices reported under ambient conditions via solution processing but also elucidates significant relationships among chemical structures, molecular properties, self-assembly from solution into a thin film, and semiconducting thin-film properties. The design rationales presented herein may open up new avenues for the development of high-electron-mobility novel electron-deficient indenofluorene and short-axis substituted donor–acceptor π-architectures via alkyl chain engineering and interface engineering.

## Introduction

N-type (electron-transporting) molecular semiconductors that are solution-processable and ambient-stable are relatively rare, particularly those that meet the criteria of high electron mobility (μ_e_ ≥ 0.5 cm^2^/(V·s)), near-zero turn-on voltage (*V*_on_ ≈ 0 V), and high current modulation (*I*_on_/*I*_off_ ≥ 10^6^) in organic field-effect transistors (OFETs) simultaneously.^1−4^ To date, only a limited number of molecular structures, which are based on naphthalene diimide (NDI), perylene diimide (PDI), and quinoidal π-architectures, have exhibited high electron mobilities in their solution-processed films under ambient conditions along with the aforementioned OFET characteristics (see Table S1 for the full list of semiconductors).^5−7^ While a delocalized π-electronic structure with a stabilized (i.e., *E*_LUMO_ < −4.0 eV) lowest unoccupied molecular orbital (LUMO) is now considered a necessity for ambient-stable electron transport,^8^ it does not necessarily guarantee a high electrical performance in n-channel OFET devices, and it tends to enhance sensitivity against electron doping from the donor sites on the dielectric surface and from the source-drain metallic contacts.^9^ Achieving a finely tuned LUMO energy level (−4.0 to −4.3 eV) is crucial for attaining a high *I*_on_/*I*_off_ ratio and minimizing *V*_on_, highlighting the importance of this parameter.^10^ On the other hand, it is also quite crucial to properly modify the dielectric surface to enable efficient electron transport in OFETs.^11,12^ Organic semiconductors typically have low surface energies due to their hydrophobic π-structures and alkyl substituents, which creates a mismatch in surface energies when they are in contact with inorganic oxide dielectric surfaces, typically leading to poor semiconductor microstructure/morphology.^13^ Moreover, charge carriers are accumulated within the first few semiconducting layers (<5 nm) adjacent to the dielectric surface, and interfacial charge traps are likely to deteriorate electron transport.^14,15^ Therefore, engineering of the dielectric–semiconductor interface^16^ offers a viable direction to high-performance n-channel OFETs under ambient conditions.^17,18^ In this regard, densely packed ultrathin (∼3–5 nm) polymer interlayer brushes, which are prepared via covalent tethering (i.e., grafting-to method) of end-functionalized hydrophobic polymer chains onto inorganic oxide dielectrics, have become an attractive interface engineering approach in the past decade.^19,20^ Specifically, following the initial report in 2010 by Lee, Cho, et al.,^21^ polystyrene (PS)-grafted oxide dielectrics have been studied with numerous p-type organic semiconductors yielding improved (4–5×) hole mobilities (e.g., μ_h_ = 0.82 cm^2^/(V·s) for pentacene^22^ and 0.84–2.10 cm^2^/(V·s) for 5,11-bis(triethylsilylethynyl)anthradithiophene^23^) as compared to conventional hydrophobic self-assembled monolayers (e.g., octadecyltrichlorosilane (OTS) or hexamethyldisilazane (HMDS)). These improvements were revealed to arise from advantageous nanostructure formation with strong π-interactions and better grain interconnectivity in the p-type semiconductor layer. Therefore, although it has been less explored, it is reasonable to expect that dense ultrathin polymer interlayers would enhance the electron mobilities of n-type semiconductors, particularly in the context of urgently needed solution-processed and ambient-stable OFETs.

**Figure 1:**
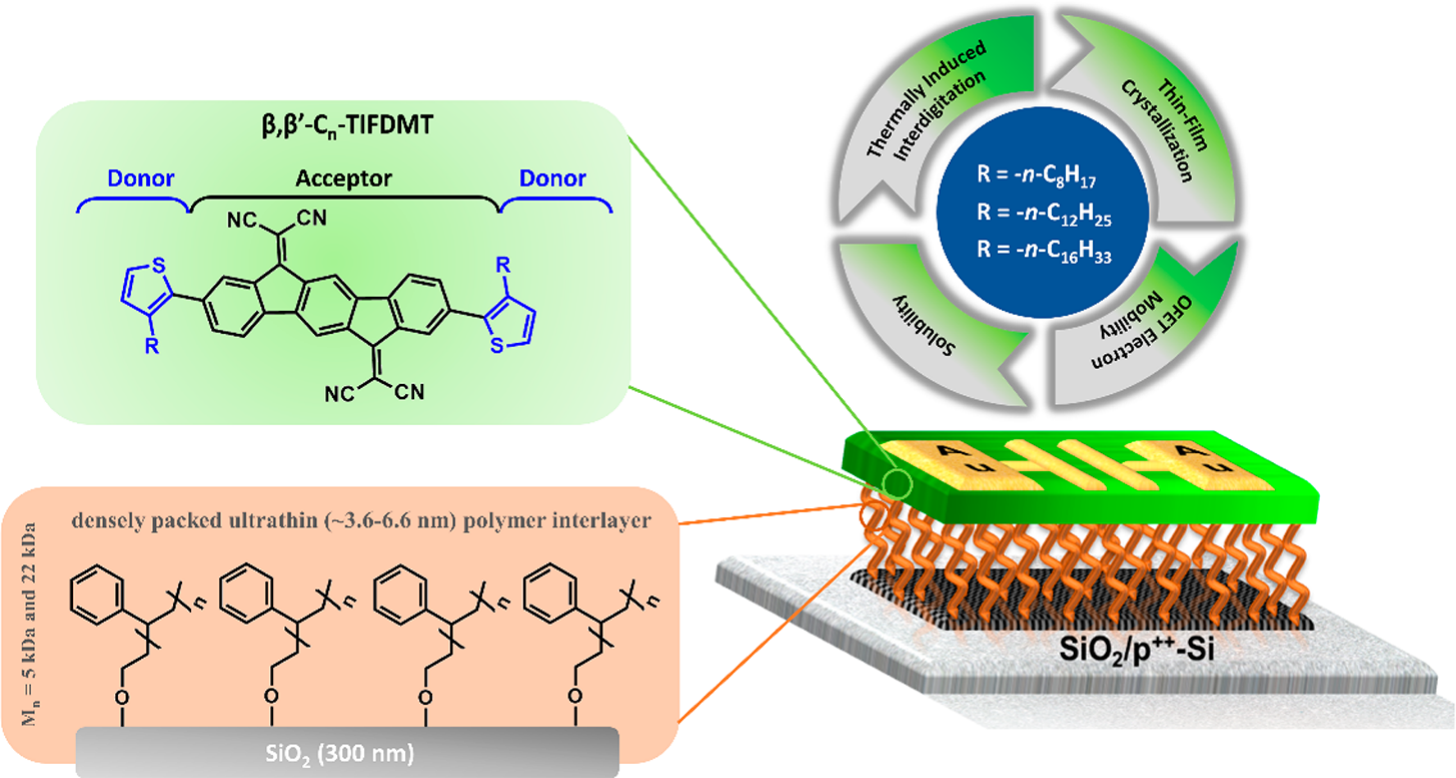
Chemical structures of solution-processable low-LUMO 2,2′-(2,8-bis(3-alkylthiophen-2-yl)indeno[1,2-*b*]fluorene-6,12-diylidene)dimalononitrile small molecules, **β,β′-C**_***n***_**-TIFDMT**s, having varied alkyl chain lengths (*n* = 8, 12, 16) and the OFET device structure (p^2+^-Si/SiO_2_/PS-brush/semiconductor/Au) employed in this study showing the PS-brush (*M*_n_ = 5 and 22 kDa)-based semiconductor–dielectric polymer interlayer.

As pioneered by several research groups in the early 2000s, π-conjugated ladder-type indenofluorene scaffolds have attracted a great deal of attention to develop electron-transporting semiconductors for OFETs in the last two decades.^24^ One effective example of such scaffolds was developed by Marks, Facchetti, et al.,^25^ which is 2,2′-(indeno[1,2-*b*]fluorene-6,12-diylidene)dimalononitrile (**IFDM**). The structure for **IFDM** is shown as the acceptor π-unit in Figure 1, in which the indeno[1,2-*b*]fluorene π-core is functionalized with electron-withdrawing dicyanovinylene moieties at the 6,12-positions. Employing this π-core, our group has previously designed and synthesized a number of low-LUMO n-type semiconductors, 2,2′-(2,8-bis((triisopropylsilyl)ethynyl)indeno[1,2-*b*]fluorene-6,12-diylidene)dimalononitrile (**TIPS-IFDM**),^26^ 2,2′-(2,8-bis(5′-(2-octyldodecyl)-2,2′-bithiophen-5-yl)indeno[1,2-*b*]fluorene-6,12-diylidene)dimalononitrile (**2OD-TTIFDMTT**),^27^ and 2,2′-(2,8-bis(5-(2-octyldodecyl)thiophen-2-yl)indeno[1,2-*b*]fluorene-6,12-diylidene)dimalononitrile (**α,ω-2OD-TIFDMT**),^28^ yet all with electron mobilities of ∼0.01–0.1 cm^2^/(V·s) (see Figure S1 for chemical structures). Among these semiconductors, the thienyl-terminated **IFDM** molecular framework, 2,2′-(2,8-bis(thiophen-2-yl)indeno[1,2-*b*]fluorene-6,12-diylidene)dimalononitrile (**TIFDMT** in Figure 1), offers an attractive donor–acceptor–donor (D-A-D) π-architecture that forms highly ordered polycrystalline microstructures in solution-processed thin films. In addition, this framework offers a viable architecture for alkyl chain engineering on the β-positions of the terminal thienyl units. While alkyl groups have been widely used to tune the solubility of π-conjugated semiconducting molecules,^29^ they have also played critical roles in determining thermal properties and thin-film molecular packing.^30−33^ The chain length, branching, and attachment position with respect to the π-core (e.g., short vs long molecular axis) have critical functions in determining crystal motifs with varied degrees of interdigitation, lamellar stacking, and π-interactions.^34,35^ Our previous study demonstrated that β-substitution is more effective than α,ω-substitution for solubility in indenofluorenes.^36^ Unlike arylene-based molecular semiconductors previously reported, where β-alkylation resulted in a marked reduction in thin-film crystallinity and charge transport,^37^ β-alkyl substitution on the **TIFDMT** D-A-D π-system promotes high crystallinity and effective electron transport. Given the significance of alkyl chain engineering and the semiconductor–dielectric interface in determining OFET performance, the aforementioned rationales led us to examine the low-LUMO **β,β′-C**_***n***_**-TIFDM** π-core with three different even-numbered linear alkyl chains of octyl (−C_8_H_17_), dodecyl (−C_12_H_25_), and hexadecyl (−C_16_H_33_) on high-density, ultrathin polymer interlayer brushes. This study has resulted in the development of two new molecular structures, **β,β′-C**_**8**_**-TIFDMT** and **β,β′-C**_**16**_**-TIFDMT** (shown in Figure 1). Together with **β,β′-C**_**12**_**-TIFDMT**, these compounds form a small n-type semiconductor library that provides a deeper understanding of the influence of β-alkyl chains on solubility, physicochemical and optoelectronic properties, thin-film microstructure and morphology, and n-channel semiconductivity. The **β,β′-C**_***n***_**-TIFDMT** π-core is studied for the first time on a high-density, ultrathin polymer-brush interlayer, which led to excellent solution-processed OFET device characteristics under ambient conditions with μ_e_ values of up to ∼0.9 cm^2^/(V·s), *I*_on_/*I*_off_ ≈ 10^7^–10^8^, and *V*_on_ ∼ 0 V.

## Experimental Section

### Materials and Methods

The reactions were conducted under nitrogen by using conventional Schlenk techniques. All chemicals were purchased from commercial sources and used as received except where noted. Silica gel with a particle size of 230–400 mesh and a pore size of 60 Å was used as the stationary phase for column chromatography. NMR measurements were conducted using a Bruker 400 spectrometer (^1^H NMR 400 MHz/^13^C NMR 100 MHz). High-resolution mass spectrum measurements were carried out on a Advion Expression CMS-L spectrometer by using the atmospheric pressure chemical ionization (MS-APCI) method or on a Bruker Daltonics (microflex LT) spectrometer by using the matrix-assisted laser desorption/ionization-time-of-flight (MALDI-TOF) method. Conventional melting temperature measurements were performed using an Electrothermal IA 9000 Series melting point apparatus. Differential scanning calorimetry (DSC) and thermogravimetric analysis (TGA) were performed on Mettler Toledo DSC822e and TGA/SDTA851e instruments, respectively. Indium and zinc standards (Mettler Toledo, Schwerzenbach, Switzerland) were used for calibration in DSC. UV–vis optical absorption measurements were performed via a Shimadzu UV-1800 spectrophotometer in solution and as spin-coated thin films on quartz. Cyclic voltammetry measurements were performed in dichloromethane on a C3 cell stand with a BASi LC Epsilon electrochemical analyzer. In the CV instrument, the counter and working electrodes were platinum (Pt) and the reference electrode was Ag/AgCl (3 M NaCl). The calibrations of all potentials were done with reference to the standard ferrocene/ferrocenium redox couple (Fc/Fc^+^: *E*_1/2_ = +0.40 V measured in the same CV measurement setup). The optimizations of the molecular geometries were carried out using density functional theory (DFT) at the B3LYP/6-31G** level with Gaussian 09.^38^

### Synthesis of 2,8-Di-3-octylthiophene-indeno[1,2-*b*]fluorene-6,12-dimalononitrile (**β,β′-C_8_-TIFDMT**)

A mixture of **β,β′-C**_**8**_**-TIFDKT** (0.21 g, 0.31 mmol) and malononitrile (0.29 g, 4.39 mmol) was dissolved in dry chlorobenzene (35.0 mL) under nitrogen and stirred at 35 °C for 15 min. Then, anhydrous pyridine (0.48 mL, 5.93 mmol) and TiCl_4_ (0.34 mL, 3.10 mmol) were added, and the reaction mixture was stirred at 110 °C for 5 h under nitrogen. The resulting reaction mixture was cooled to room temperature, and it was quenched with water and extracted with chloroform. The organic phase was washed with water, dried over Na_2_SO_4_, filtered, and evaporated to dryness to give a crude product, which was purified by column chromatography on silica gel using CHCI_3_:hexanes (8:2 (v/v)) as the eluent. Finally, the product was washed with methanol and filtered to afford the final pure product as a dark green solid (0.114 g, 48% yield). ^1^H NMR (CDCl_3_): δ 8.57 (s, 2H), 8.50 (s, 2H), 7.68 (d, 2H, *J* = 8.0 Hz), 7.63 (d, 2H, *J* = 8.0 Hz), 7.31 (d, 2H, *J* = 4.0 Hz), 7.03 (d, 2H, *J* = 4.0 Hz) 2.72 (t, 4H, *J* = 8.0 Hz), 1.27 (t, 24H,), 0.86 (t, 6H, *J* = 12.0 Hz) ppm. ^13^C NMR (CDCl_3_): 14.1, 22.7, 29.0, 29.3, 29.4, 29.5, 30.8, 31.8, 78.6, 112.6, 113.1, 118.2, 121.2, 124.9, 127.5, 130.0, 134.4, 135.5, 135.8, 137.1, 139.3, 140.3, 143.4, 159.7 ppm. Mp: 237–238 °C. MS (MALDI-TOF) *m*/*z* (M^+^): calcd for C_50_H_46_S_2_N_4_, 767.32; found, 767.156. Anal. Calcd for C_50_H_46_S_2_N_4_: C, 78.29; H, 6.04; N, 7.30. Found: C, 78.42; H, 6.17; N, 7.52.

### Synthesis of 2,8-Di-3-dodecylthiophene-indeno[1,2-*b*]fluorene-6,12-dimalononitrile (**β,β′-C_12_-TIFDMT**)

A mixture of **β,β′-C**_**12**_**-TIFDKT** (0.28 g, 0.36 mmol) and malononitrile (0.33 g, 5.00 mmol) was dissolved in dry chlorobenzene (35.0 mL) under nitrogen and stirred at 35 °C for 15 min. Then, anhydrous pyridine (0.49 mL, 6.06 mmol) and TiCl_4_ (0.39 mL, 3.56 mmol) were added, and the reaction mixture was stirred at 110 °C for 5 h under nitrogen. The resulting mixture was allowed to cool to room temperature, and it was quenched with water and extracted with chloroform. The organic phase was washed with water, dried over Na_2_SO_4_, filtered, and evaporated to dryness to give a crude product, which was purified by column chromatography on silica gel using CHCI_3_:hexanes (8:2 (v/v)) as the eluent. Finally, the product was washed with methanol and filtered to afford the final pure product as a dark green solid (0.161 g, 51% yield). ^1^H NMR (CDCl_3_): δ 8.61 (s, 2H), 8.52 (s, 2H), 7.70 (d, 2H, *J* = 8.0 Hz), 7.64 (d, 2H, *J* = 8.0 Hz), 7.31 (d, 2H, *J* = 4.0 Hz), 7.03 (d, 2H, *J* = 4.0 Hz) 2.70 (t, 4H, *J* = 8.0 Hz), 1.24 (t, 40H), 0.85 (t, 6H, *J* = 12.0 Hz) ppm. ^13^C NMR (CDCl_3_): 14.1, 22.7, 29.0, 29.3, 29.4, 29.5, 30.8, 31.8, 78.6, 112.6, 113.1, 118.2, 121.2, 124.9, 127.5, 130.0, 134.4, 135.5, 135.8, 137.1, 139.3, 140.2, 143.1, 159.6 ppm. Mp: 231–232 °C. MS (MALDI-TOF) *m*/*z* (M^+^): calcd for C_58_H_62_S_2_N_4_, 878.44; found, 879.029. Anal. Calcd for C_58_H_62_S_2_N_4_: C, 79.23; H, 7.11; N, 6.37. Found: C, 79.44; H, 7.21; N, 6.56.

### Synthesis of 2,8-Di-3-hexadecylthiophene-indeno[1,2-*b*]fluorene-6,12-dimalononitrile (**β,β′-C_16_-TIFDMT**)

A mixture of **β,β′-C**_**16**_**-TIFDKT** (165 mg, 0.18 mmol) and malononitrile (170 mg, 2.57 mmol) was dissolved in dry chlorobenzene (25.0 mL) under nitrogen and stirred at 35 °C for 15 min. Then, anhydrous pyridine (0.30 mL, 3.71 mmol) and TiCl_4_ (0.20 mL, 1.82 mmol) were added, and the reaction mixture was stirred at 110 °C for 5 h under nitrogen. The resulting mixture was allowed to cool to room temperature, and it was quenched with water and extracted with chloroform. The organic phase was washed with water, dried over Na_2_SO_4_, filtered, and evaporated to dryness to give a crude product, which was purified by column chromatography on silica gel using CHCl_3_:hexanes (8:2 (v/v)) as the eluent. Finally, the product was washed with methanol and filtered to afford the final pure product as a dark green solid (64.3 mg, 35% yield). ^1^H NMR (CDCl_3_): δ 8.61 (s, 2H), 8.53 (s, 2H), 7.70 (d, 2H, *J* = 8.0 Hz), 7.64 (d, 2H, *J* = 8.0 Hz), 7.31 (d, 2H, *J* = 4.0 Hz), 7.03 (d, 2H, *J* = 4.0 Hz) 2.70 (t, 4H, *J* = 4.0 Hz), 1.24 (t, 56H,), 0.87 (t, 6H, *J* = 12.0 Hz) ppm. ^13^C NMR (CDCl_3_): 14.1, 22.7, 29.0, 29.4, 29.7, 30.8, 31.9, 78.8, 112.8, 118.2, 120.5, 121.2, 122.5, 124.8, 127.5, 130.0, 134.8, 135.9, 136.9, 137.1,140.2, 143.3, 159.6 ppm. Mp: 221–222 °C. MS (MALDI-TOF) *m*/*z* (M^+^): calcd for C_66_H_78_S_2_N_4_, 990.57; found, 991.598. Anal. Calcd for C_66_H_78_S_2_N_4_: C, 79.95; H, 7.93; N, 5.65. Found: C, 79.85; H, 8.02; N, 5.79.

### Solubility Measurements

The solubility of the current semiconductors was measured by using a gravimetric method. A small amount (2.0–3.0 mg) of organic semiconductor solid was precisely weighed into a vial, and incremental volumes (in 50–100 μL portions) of chloroform were added via micropipet. After each addition, the solution was stirred/sonicated for 10–20 min at room temperature and heated to ∼50 °C to kinetically aid the dissolution process. The solution was cooled to room temperature for visual observation, and solvent addition was continued until complete dissolution was visually confirmed at room temperature. The solubility was calculated based on the initial semiconductor solid weight (*m*_semiconductor_) and the total amount of solvent (*V*_solvent_) used based on the equation solubility = *m*_semiconductor_/*V*_solvent_. The final completely dissolved semiconductor solution was filtered through a PTFE syringe filter (VWR, part of Avantor, 0.45 μm pore size) and then evaporated to dryness with a rotary evaporator. The recovered semiconductor was weighed again in order to double check the dissolved semiconductor amount in *V*_solvent_; the difference between the originally weighed and the recovered semiconductor solids was typically less than 3–4%.

### PS-Brush Interlayer Fabrication

A heavily p-doped Si wafer with a thermally grown 300 nm thick SiO_2_ layer was used as the substrate. The substrates were cleaned in an ultrasonic bath by using hexane, acetone, and ethanol for 10 min, respectively. After the cleaning process, the substrates were dried under N_2_ flow. Next, air plasma treatment (Harrick Plasma Cleaner) for 3 min was applied to the substrates to activate their surfaces. The air plasma treated SiO_2_ layer was modified by using hydroxyl-terminated polystyrene (PS-OH) (Polymer Source Inc., Canada) with molecular weights of *M*_n_ = 5 kDa and *M*_n_ = 22 kDa via a “grafting-to” method. In this method, PS-OH in a 0.5 wt % toluene solution was spin-coated on top of the SiO_2_ layer, followed by a thermal treatment at 170 °C for 48 h in a vacuum oven. This annealing process allowed PS-OH chains to tether covalently from their hydroxyl end groups to the hydroxylated SiO_2_ dielectric surface. After annealing, PS-brush-treated substrates were cleaned in an ultrasonic bath by using toluene in order to remove any free PS-OH chains. The contact angle measurement was carried out for the PS-brush-treated substrate with a Biolin Scientific Attension Theta Lite instrument. The thickness measurements for the PS-brush layers were performed using an ellipsometer (Gaertner, LSE 2085–AK), and the surface morphology was evaluated by atomic force microscopy (NanoSurf, FlexAFM C3000). The UV–vis diffuse reflectance spectra of the semiconductor thin films on the PS-brush interlayer were collected on a Shimadzu UV-3600i Plus spectrophotometer using an ISR-603 integrating sphere.

### OFET Device Fabrication and Electrical Characterization

Semiconducting thin films (∼40–45 nm) were deposited onto the PS-brush-treated substrates by spin-coating the corresponding **β,β′-C**_***n***_**-TIFDMT** solution in CHCl_3_ (4.0 mg/mL) at 1100 rpm under ambient conditions, followed by thermal annealing at 100, 120 and 150 °C (for 30 min) in a vacuum oven. The OFET device structure was completed with thermal evaporation of 50 nm thick Au source-drain electrodes (growth rate 0.2 Å/s) to give channel lengths (*L*) and widths (*W*) of 30, 40, 50, 60, 80, and 1000 μm, respectively. The electrical characteristics of the OFETs were measured under ambient conditions (without excluding natural or fluorescent lighting) using a Keithley 2614B source-measure unit in an Everbeing BD-6 probe station. The field effect mobility was calculated from the *I*_SD_^1/2^ vs *V*_G_ plot by using an equation derived for the saturation region
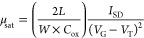
1where *I*_SD_ is the source-drain current, *V*_G_ and *V*_T_ are the gate and threshold voltages, respectively, and *C*_ox_ is the specific capacitance of the gate dielectric with PS-brush interlayer per unit area (taken as 10.5 nF/cm^2^).^22,23^ The surface morphologies and microstructures of the semiconductor thin films were studied by atomic force microscopy on a NanoSurf FlexAFM C3000 instrument, and by grazing incidence X-ray diffraction (GIXRD) on a Malvern Panalytical Empyrean diffractometer. A confocal Raman spectrometer (Jasco NRS-4500) with an excitation wavelength of 785 nm was used for the Raman investigation of semiconductor thin films. All Raman spectra were collected using a 20× microscope objective, an optical power of 75 mW, and an acquisition time of 60 s.

## Results and Discussion

### Semiconductors Synthesis and Characterization

**Scheme 1:**
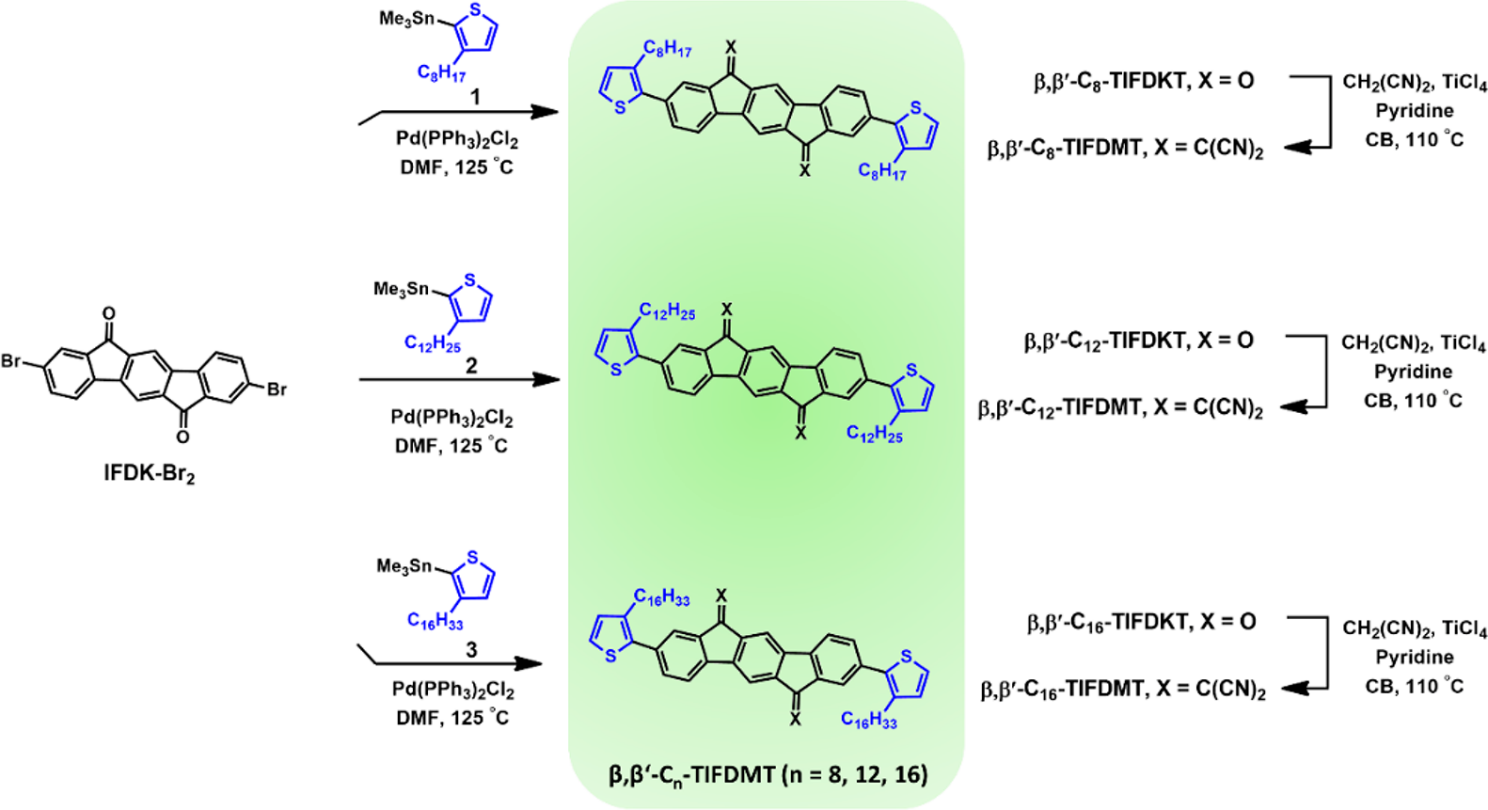
Synthesis of 2,2′-(2,8-Bis(3-alkylthiophen-2-yl)indeno[1,2-*b*]fluorene-6,12-diylidene)dimalononitrile Small Molecules **β,β′-C**_**8**_**-TIFDMT**, **β,β′-C**_**12**_**-TIFDMT**, and **β,β′-C**_**16**_**-TIFDMT**

Scheme 1 illustrates the synthesis of 2,2′-(2,8-bis(3-alkylthiophen-2-yl)indeno[1,2-*b*]fluorene-6,12-diylidene)dimalononitrile small molecules **β,β′-C**_***n***_**-TIFDMT**s. The dibrominated indeno[1,2-*b*]fluorene-6,12-dione π-core, **IFDK-Br**_**2**_, is a crucial intermediate for producing these molecules and was obtained via a two-step synthesis (Scheme S1; see the Supporting Information for synthesis details) in accordance with our group’s previously reported procedure.^36^ The (3-alkylthiophen-2-yl)trimethylstannane reagents **1**–**3** were synthesized for each molecule with a different alkyl chain (−C_*n*_H_2*n*+1_, *n* = 8, 12, and 16), using a synthetic route involving Kumada coupling, bromination, and stannylation steps (Scheme S1; see the Supporting Information for synthesis details). **IFDK-Br**_**2**_ was reacted with the corresponding reagents **1**–**3** following a Pd(PPh_3_)_2_Cl_2_-catalyzed Stille cross-coupling protocol in DMF at 125 °C. A highly polar solvent at an elevated temperature was used in these cross-coupling reactions in order to partially solubilize **IFDK-Br**_**2**_. Note that **IFDK-Br**_**2**_ exhibits a very low solubility in common organic solvents. 2,8-Bis(3-alkylthiophen-2-yl)indeno[1,2-*b*]fluorene-6,12-dione compounds **β,β′-C**_**8**_**-TIFDKT**, **β,β′-C**_**12**_**-TIFDKT**, and **β,β′-C**_**16**_**-TIFDKT** were obtained in 9–45% yields. Finally, these dicarbonyl-functionalized indenofluorene compounds underwent a Knoevenagel condensation reaction with malononitrile in chlorobenzene in the presence of a pyridine base and TiCl_4_ Lewis acid to yield the target compounds **β,β′-C**_**8**_**-TIFDMT**, **β,β′-C**_**12**_**-TIFDMT**, and **β,β′-C**_**16**_**-TIFDMT** in 35–51% yields. The molecular solids were soluble in common nonprotic organic solvents, which allowed for convenient chromatographic purifications. The chemical structures and the purities were confirmed via ^1^H and ^13^C NMR (Figures S2, S4–S9, S11–S16, and S18–S23), elemental analysis, and mass spectrometry (atmospheric-pressure chemical ionization (APCI) and matrix-assisted laser desorption/ionization (MALDI-TOF) techniques) (Figures S3, S10, S17, and S24).

### Effects of Alkyl Chains on Solubility, Physicochemical, and Optoelectronic Properties

**Figure 2:**
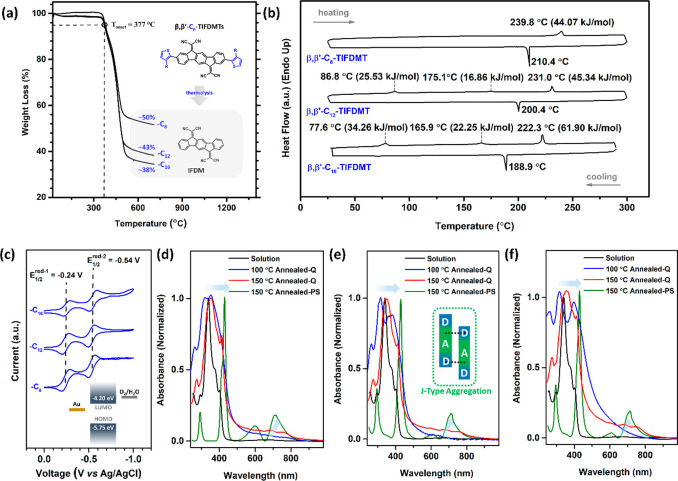
(a) Thermogravimetric analysis and (b) differential scanning calorimetry scans of **β,β′-C**_**8**_**-TIFDMT**, **β,β′-C**_**12**_**-TIFDMT**, and **β,β′-C**_**16**_**-TIFDMT** at a temperature ramp of 10 °C min^–1^ under N_2_. The inset in the TGA shows the final mass percentages after thermolysis and the proposed thermolysis reaction. (c) Cyclic voltammograms of the present **β,β′-C**_***n***_**-TIFDMT** molecules (vs Ag/AgCl (3 M NaCl)) in dichloromethane (0.1 M Bu_4_N^+^PF_6_^–^, scan rate 50 mV/s) measured for different alkyl lengths (−C_*n*_, *n* = 8, 12, and 16). The inset shows an energy diagram of experimentally estimated HOMO/LUMO energy levels with respect to the Au Fermi level and the O_2_/H_2_O electrochemical redox couple^41^ at the air–thin film interface. Optical absorption spectra of **β,β′-C**_**8**_**-TIFDMT** (d), **β,β′-C**_**12**_**-TIFDMT** (e), and **β,β′-C**_**16**_**-TIFDMT** (f) in dichloromethane (∼1 × 10^–5^ M) (black lines) and as spin-coated thin films on quartz after 100 °C (blue lines) and 150 °C (red lines) thermal annealing (for 30 min under vacuum). For spin-coated **β,β′-C**_***n***_**-TIFDMT** thin films on the p^++^-Si/SiO_2_/PS-brush (*M*_n_ = 5 kDa) substrate (after 150 °C themal annealing), the collected diffuse reflectance data are transformed to pseudoabsorption data (green lines) using the Kubelka–Munk function.^45,46^ A schematic of a typical slipped-stacked J-aggregate interaction is shown in the inset of (e).

The thermal properties of the present **β,β′-C**_***n***_**-TIFDMT**s were investigated by using thermogravimetric analysis (TGA), differential scanning calorimetry (DSC), and conventional melting point measurements. As shown in Figure 2a, excellent thermal stabilities with thermolysis onset (5% mass loss) temperatures of 377 °C were observed for all three molecules. The final thermolysis mass losses were calculated as ∼50% for C_8_, ∼57% for C_12_, and ∼62% for C_16_, which were attributed to the thermal decomposition of alkylthienyl end units from the **β,β′-C**_***n***_**-TIFDMT** compounds (Figure S25). This yielded the IFDM π-core as the final solid. On the other hand, sharp thermal transitions of solid–isotropic liquid origin were observed by using a conventional melting point apparatus. The melting temperature (*T*_mp_) was found to gradually decrease as the alkyl chain length increased (*T*_mp_ = 237–238 °C → 231–232 °C → 221–222 °C for C8 → C12 → C16). These melting transitions, along with some prior endothermic transitions, were evident in the DSC scans (Figure 2b), yet with an opposite trend in the corresponding total enthalpy changes (Δ*H*_S→I_) for solid–isotropic liquid transitions. In sharp contrast to the observed melting temperature trend, Δ*H*_S→I_ was found to increase with the alkyl chain length as 44.07 kJ/mol for C_8_, 87.73 kJ/mol for C_12_, and 118.41 kJ/mol for C_16_ (Table S2). Since D-A-D π-backbone structural properties (i.e., π-coplanarities with θ_IFDM-Thienyl_ ≈ 47°)^36^ are expected to be the same in all three molecules, the observed Δ*H*_S→I_ increase with alkyl length is attributed to enhanced solid-state cohesive energetics via dispersion interactions between extended methylene units (−(CH_2_)_*n*_–CH_3_, *n* = 7 → 11 → 15).^39,40^ The observed endothermic transitions for C_12_ and C_16_ prior to melting suggest the presence of a liquid crystal phase (i.e., probably smectic)^41^ in certain temperature ranges. The relationship between solubility and cohesive energetics has been initially established by Hildebrand and Scatchard,^42,43^ and solubility is correlated with cohesive energetics based on both ideal and regular solution theories.^42^ The solubilities of the present molecules were measured by using a gravimetric method, which closely follow the corresponding total enthalpy changes.^30,40^ While **β,β′-C**_**8**_**-TIFDMT** exhibits a solubility of 10.3 mg/mL (0.0134 M) in chloroform at room temperature, **β,β′-C**_**12**_**-TIFDMT** and **β,β′-C**_**16**_**-TIFDMT** exhibit decreased (∼2–6×) solubilities of 6.9 mg/mL (7.85 × 10^–3^ M) and 2.5 mg/mL (2.52 × 10^–3^ M), respectively. A similar solubility dependence on alkyl chain length and cohesive energetics was previously demonstrated for fused thienoacene compounds.^32,40^ It is noteworthy that the completely dissolved solution of **β,β′-C**_**16**_**-TIFDMT** at concentrations of >3 mg mL^–1^ starts to aggregate upon prolonged waiting (>2–3 h), probably through enhanced dispersion interactions between relatively long linear −C_16_H_33_ chains. Therefore, thin-film processing (*vide infra*) for **β,β′-C**_**16**_**-TIFDMT** was performed by using slightly heated solutions (∼40–50 °C). On the other hand, for complete dissolution of **β,β′-C**_**12**_**-TIFDMT**, heating at ∼40–50 °C was required; however, the solid remains in solution upon cooling to room temperature. This suggests that the thermal treatment in the case of **β,β′-C**_**12**_**-TIFDMT** kinetically drives the initial dissolution process for better solvent–solute interactions, rather than a pure thermodynamic effect.^42^ Because Δ*H*_S→I_ increases and *T*_mp_ decreases with the increased alkyl length, the corresponding total entropy changes for solid–isotropic liquid transitions also showed clear increases (Δ*S*_S→I_ = 85.91 → 198.47 → 273.3 J/(K mol) for C_8_ → C_12_ → C_16_, Table S2; see the Supporting Information for further details). This entropy increase is undoubtedly endorsed by enhanced degrees of freedom (*gauche* and *trans* conformations)^44^ for long alkyl substituents via internal bond rotations in the melted state, adding an entropy value of ∼10 J/(K mol) for each additional methylene unit, which is consistent with earlier studies.^30,40^ For each alkyl substituent, the **β,β′-C**_***n***_**-TIFDMT** molecule exhibits an ∼70–80 °C increased melting temperature compared to the corresponding **β,β′-C**_***n***_**-TIFDKT** molecule. This reflects enhanced intermolecular interactions through increased local dipoles, greater π-delocalization, and increased D-A-D electronic structure going from carbonyl (C=O) to the dicyanovinylene unit (C=C(CN)_2_).

The optical and electrochemical characterizations of the present molecules were performed by UV–vis absorption/diffuse reflectance spectroscopy and cyclic voltammetry (CV). As shown in Figure 2c, all molecules exhibit two reversible reduction peaks in dichloromethane solution in the voltage range of 0 to −1.0 V (vs Ag/AgCl (3 M NaCl)). The reversibility indicates the excellent redox stability of the present **β,β′-C**_***n***_**-TIFDMT** molecules.^47^ The first and second half-wave reduction potentials (*E*_1/2_^red-1,2^) are located at low voltages of −0.24 and −0.54 V, respectively. On the other hand, one main optical absorption peak at 342 nm, along with an extremely weak peak at 656 nm, was observed in dichloromethane solutions (black lines in Figure 2d–f). The molar extinction coefficient was calculated as 1.02 × 10^5^ M^–1^ cm^–1^ for −C_8_, which gradually decreases to 5.85 × 10^4^ and 1.32 × 10^4^ M^–1^ cm^–1^ with longer chains of −C_12_ and −C_16_, respectively. Two lower energy shoulders to the main peak were evident at 377 and 404 nm (∼0.20–0.23 eV peak intervals), which corresponds to a vibronic coupling with the C=C stretching modes^41^ and originates from the rigid TIFDMT π-backbone. The optical band gaps in solution are estimated to be 1.55 eV from the low-energy band edges. From the CV and UV–vis data, the HOMO/LUMO energy levels are estimated to be −5.75–-4.20 eV for the present **β,β′-C**_***n***_**-TIFDMT** molecules, which appear to be minimally affected by the length of the alkyl chain.

Going from solution to spin-coated thin films on quartz, for 100 °C annealed **β,β′-C**_***n***_**-TIFDMT** thin films (blue lines in Figure 2d–f), the main absorption peaks broaden and show two vibronically featured maxima with a red and blue shift of Δλ = ±20–45 nm with respect to those in solution. This is attributed to kinetically driven aggregation behavior of the present molecules during the fast spin-coating process forming structurally diverse aggregate states (*vide infra*; see microstructural/morphological characterizations of the 100 °C annealed thin films).^48^ When the thermal annealing temperature was raised to 150 °C (red lines in Figure 2d–f), the absorption peaks get narrower and shift to lower energies (Δλ ≈ +30 nm), showing a sharp shoulder peak at 410–415 nm. Also, the weak absorption bands at 656 nm shift to the 700–800 nm region with increased intensities. These spectral changes indicate a favored solid-state ordering with enhanced intermolecular interactions in spin-coated thin films after 150 °C thermal annealing. The tendency of the present **β,β′-C**_***n***_**-TIFDMT** molecules to form J-aggregates was evident in the 150 °C annealed films on quartz, which became more profound when thin films were deposited on highly dense polymer interlayers. The UV–vis diffuse reflectance spectra collected for **β,β′-C**_***n***_**-TIFDMT** thin films (Figure S26) on the p^++^-Si/SiO_2_/PS-brush (*M*_n_ = 5 kDa) substrate are transformed to pseudoabsorption data using the Kubelka–Munk function.^46^ Amazingly, these absorption spectra (green lines in Figure 2d–f) showed no evident molecular transitions and exhibited completely new red-shifted (Δλ_max_ = +85–90 nm), narrow absorptions at λ_max_ = 427–430 nm (fwhm ≈ 20 nm). These spectral changes clearly point at the formation of J-aggregation on the PS-brush interlayer, in which the D-A-D π-frameworks are in a slipped-stacked arrangement.^49,50^ This arrangement facilitates favorable intermolecular D···A interactions between thienyl (donor) and IFDM (acceptor) units, which also manifests itself in the significantly enhanced, broad low-energy HOMO → LUMO transitions at ∼600–800 nm. The structural ordering in the solid state on the PS-brush interlayer is consistent with the thin-film XRD and AFM findings (*vide infra*).

### Preparation and Characterization of High-Density PS-Brush Interlayers

**Figure 3:**
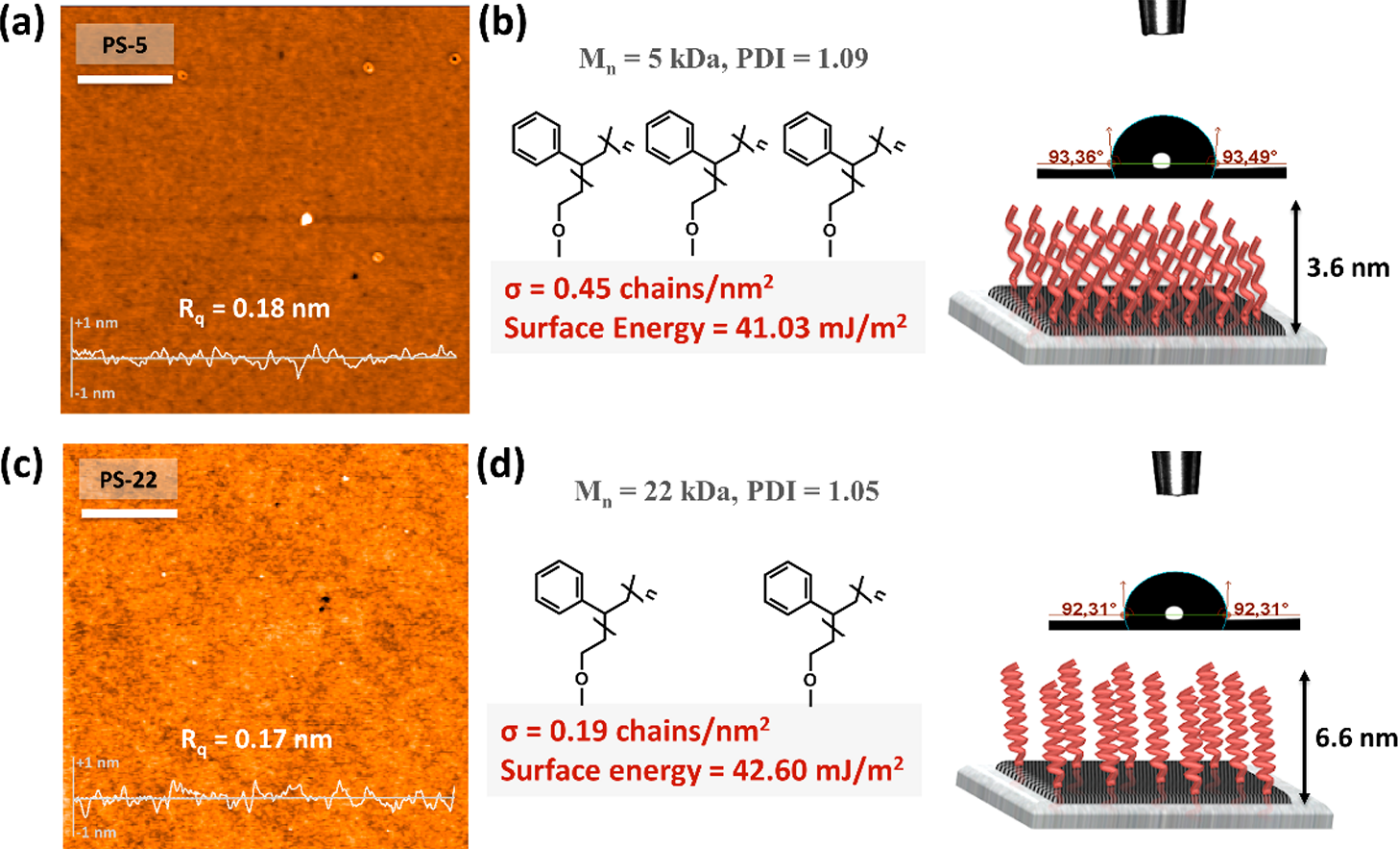
(a, c) Atomic force microscopy (AFM) topography images and cross-sectional AFM height profiles along with root-mean-square roughnesses (*R*_q_s) for p^++^-Si/SiO_2_ (300 nm)/PS-brush (*M*_n_ = 5 or 22 kDa) surfaces. (b, d) Water contact angle measurements and schematic of different grafting densities for PS-5 and PS-22 polymer brushes with the corresponding surface energies and grafting densities (σ’s). Scale bars denote 2 μm.

We employed hydroxyl-end-functionalized PS polymers (PS-OH) with relatively low molecular weights (*M*_n_ = 5 and 22 kDa) in our study, and the PS-brush interlayers were prepared following a “grafting-to” methodology.^21^ This is because high-molecular-weight (*M*_w_ > 100 kDa) polymers have been reported to yield a low areal grafting density (σ ≪ 0.1 chains/nm^2^) with pancake-like polymer interlayer structures, which can impede the semiconductor molecular self-assembly, leading to an unfavorable packing/microstructure for efficient charge transport.^23^ Both PS-5 and PS-22 surfaces are found to be morphologically very smooth and pinhole-free, showing a low root-mean-square (*R*_q_) roughness value of 0.17–0.18 nm for a 10 × 10 μm^2^ area (Figure 3a,c). A low *R*_q_ value is essential because a roughness larger than ∼1–2 nm, which is on the order of the thickness of a few semiconducting layers, could interfere with the molecular self-assembly and create local charge carrier trap sites.^51^ On the other hand, both surfaces were found to be hydrophobic with large water contact angle values of 93.0 ± 0.5° (for PS-5, Figure 3b inset) and 92.0 ± 0.6° (for PS-22, Figure 3d inset). The surface energies were measured to be 41.03 and 42.60 mJ/m^2^, respectively. These surface energies were calculated by measuring contact angles and surface tensions of different liquids (i.e., distilled water, diiodomethane, formamide, and ethylene glycol) according to Wu’s method,^52^ and they are much lower than that of the silanol surface (γ > 87 mJ/m^2^). The areal grafting density (σ) is the key parameter when considering the surface coverage and the positional arrangement of polymer brushes with respect to each other (e.g., brush vs mushroom-like). It can be calculated by using the equation
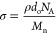
2where ρ is the polymer mass density (ρ_PS_ = 1.05 g/cm^3^),^23^*d*_o_ is the brush height (polymer interlayer film thickness), and *N*_A_ and *M*_n_ represent Avogadro’s constant and the number-average molecular weight of the polymer, respectively. Based on the polymer interlayer film thicknesses (*d*_o_ = 3.6 ± 0.2 nm for PS-5 and *d*_o_ = 6.6 ± 0.2 nm for PS-22) measured via ellipsometry, excellent grafting densities of 0.45 and 0.19 chains/nm^2^ were calculated for PS-5 and PS-22, respectively. These high densities (σ > 0.1 chains/nm^2^), along with low surface energy and smoothness, could hamper any undesired molecular diffusion into the polymer interlayer, which in turn can minimize any microstructural or morphological disorders at the semiconductor–dielectric interface (*vide infra*). Amazingly, the surface prepared in this study by the lower-molecular-weight (*M*_n_ = 5 kDa) chains show one of the finest grafted-PS-brush arrangements of those given in the literature,^22^ and it offers a highly favorable dielectric surface for **β,β′-C**_***n***_**-TIFDMT** depositions.

### OFET Fabrication and Characterization under Ambient Conditions

**Figure 4:**
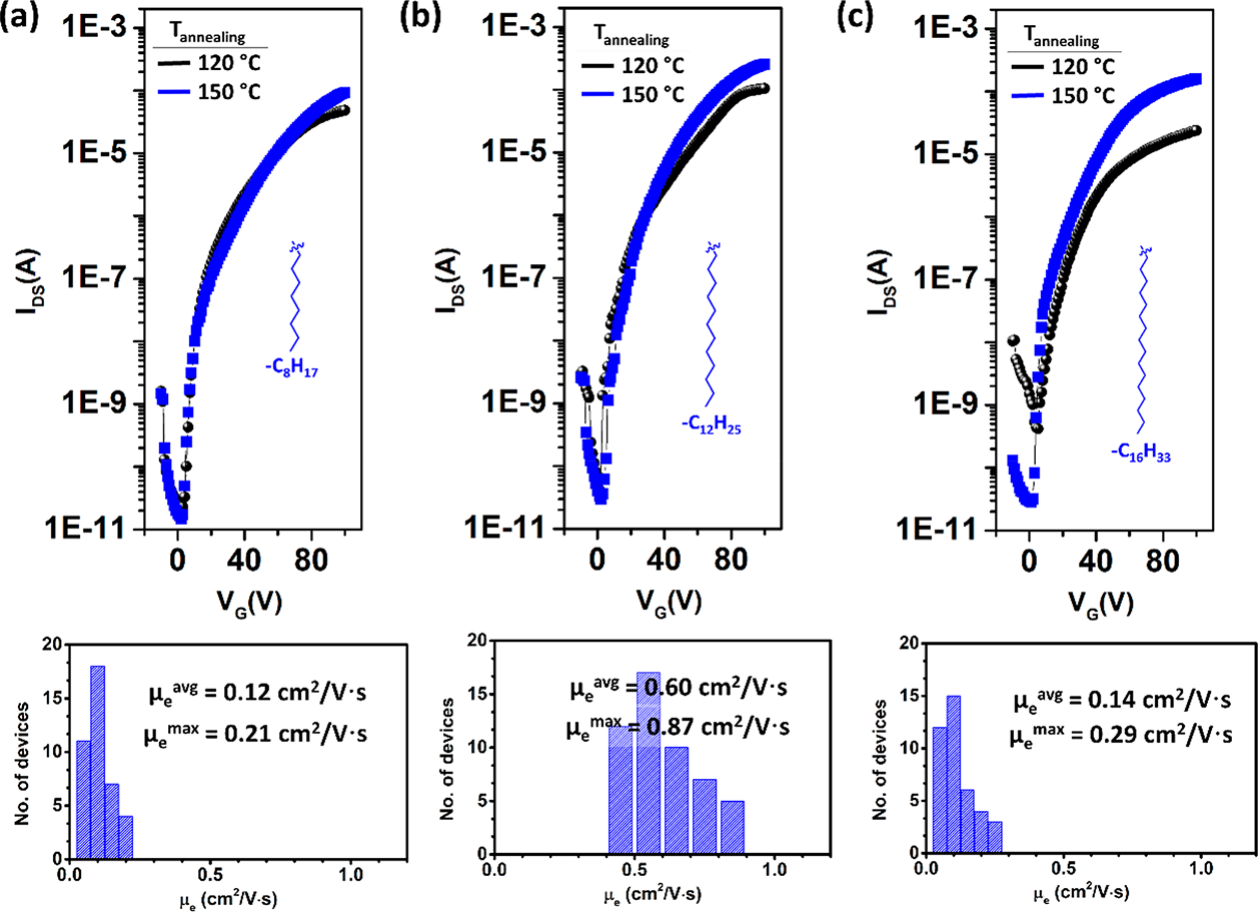
Transfer plots (*V*_DS_ = 100 V) measured under ambient conditions for p^++^-Si/SiO_2_/PS-brush (*M*_n_ = 5 kDa)/semiconductor/Au OFET devices based on the semiconductor molecules (a) **β,β′-C**_**8**_**-TIFDMT**, (b) **β,β′-C**_**12**_**-TIFDMT**, and (c) **β,β′-C**_**16**_**-TIFDMT** and the corresponding statistical distribution of μ_e_ values based on the number of measured devices (total >40) for 150 °C annealed OFETs (given under each transfer curve). The annealing temperature for each device is given in the transfer plots.

The semiconductor properties of the current **β,β′-C**_***n***_**-TIFDMT**s were studied in bottom-gate/top-contact (BG/TC) OFETs by spin-coating (Figure 1). In order to investigate thermally induced microstructural ordering and thin-film crystallization on the PS-brush interlayer, the **β,β′-C**_***n***_**-TIFDMT** thin films (∼40–45 nm) were annealed at different temperatures of 100, 120, and 150 °C. Higher annealing temperatures were found to have negligible effects. As a result of their highly stabilized LUMOs, all **β,β′-C**_***n***_**-TIFDMT** molecules functioned as n-type semiconductors under ambient conditions with excellent electron mobilities of as high as 0.21 cm^2^/(V·s) (μ_e_^avg^ = 0.12 cm^2^/(V·s)) for **β,β′-C**_**8**_**-TIFDMT**, 0.87 cm^2^/(V·s) (μ_e_^avg^ = 0.60 cm^2^/(V·s)) for **β,β′-C**_**12**_**-TIFDMT**, and 0.29 cm^2^/(V·s) (μ_e_^avg^ = 0.14 cm^2^/(V·s)) for **β,β′-C**_**16**_**-TIFDMT**. Typical transfer and output plots are shown in Figure 4 and Figure S27, respectively. In addition to high electron mobilities, excellent current modulation characteristics with very large *I*_on_/*I*_off_ values of 10^7^–10^8^ and near-zero turn-on voltages were realized in our solution-processed OFETs. This is attributed to the well-tuned LUMO energy level (−4.20 eV) of the **TIFDMT** π-system, which, while providing ambient stability for electron transport, impedes undesired semiconductor electron doping in the off state.^9^ While **β,β′-C**_**8**_**-TIFDMT**-based OFETs showed similar n-channel semiconductivities with μ_e_^avg^ values of 0.10–0.12 cm^2^/(V·s) at 120 and 150 °C annealing temperatures (Figure 4a), respectively, **β,β′-C**_**12**_**-TIFDMT**- and **β,β′-C**_**16**_**-TIFDMT**-based OFETs showed clear enhancements as μ_e_^avg^ = 0.17 cm^2^/(V·s) → 0.60 cm^2^/(V·s) (Figure 4b) and 0.03 cm^2^/(V·s) → 0.14 cm^2^/(V·s) (Figure 4c), respectively, with increased temperature. For all molecules, OFETs with the semiconductor layer annealed at temperatures ≤100 °C showed poor n-channel activity (μ_e_^avg^ < 10^–3^ cm^2^/(V·s)).

### Morphological and Microstructural Analyses of Semiconductor Thin Films

**Figure 5:**
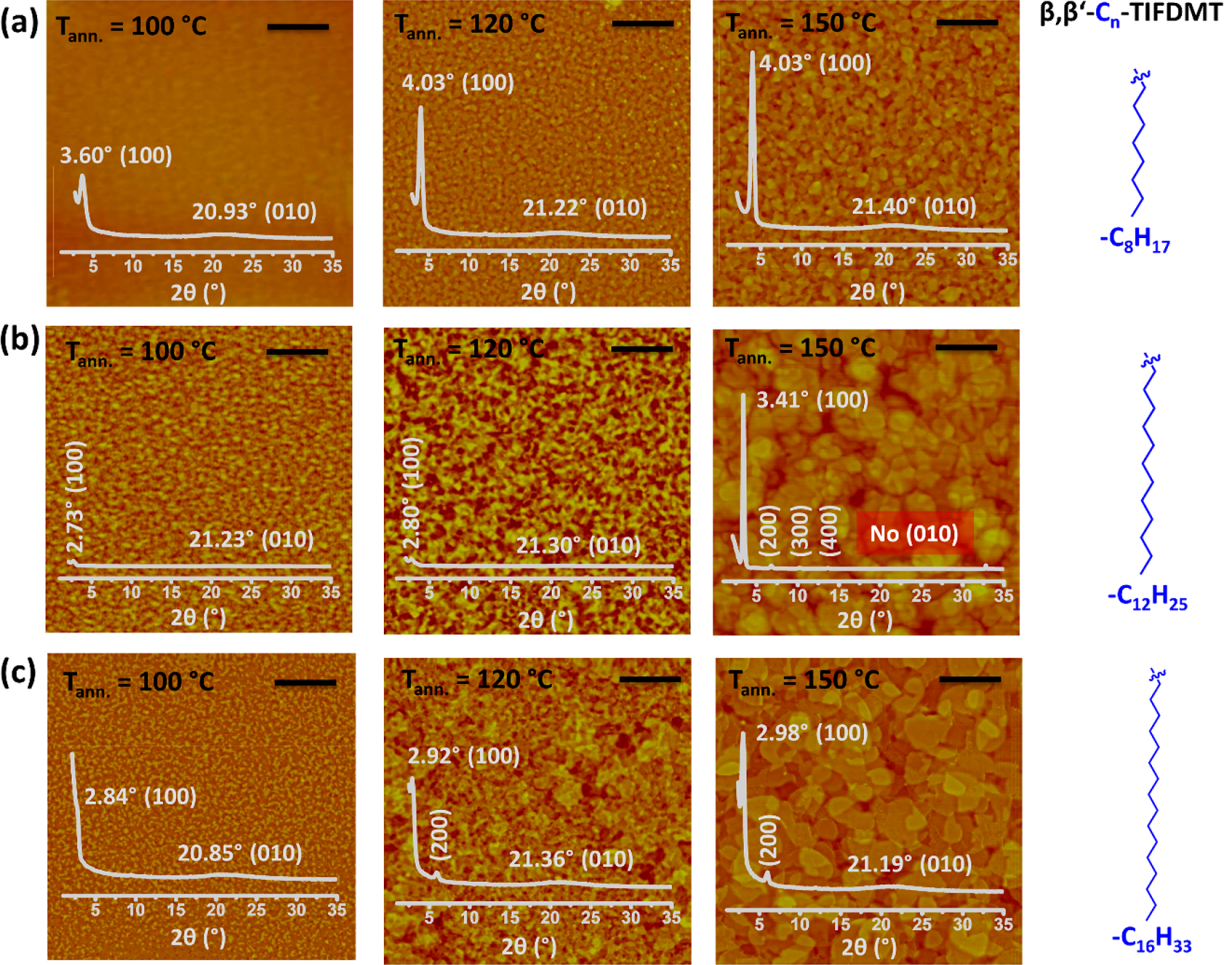
Top-view atomic force microscopy (AFM) topography images for spin-coated (a) **β,β′-C**_**8**_**-TIFDMT**, (b) **β,β′-C**_**12**_**-TIFDMT**, and (c) **β,β′-C**_**16**_**-TIFDMT** thin films on p^++^-Si/SiO_2_/PS-brush (*M*_n_ = 5 kDa) after thermal annealing at varied temperatures of 100, 120, and 150 °C. Scale bars denote 2 μm. The inset in each AFM image shows the corresponding out-of-plane grazing-incidence X-ray diffraction (GIXRD) pattern (θ_incident_ = 0.5°) with the assigned peaks and crystallographic planes ((100), (200), (300), (400), and (010)). For each semiconductor, the diffraction intensities are not normalized to display microstructural changes upon thermal annealing.

The morphologies and microstructures for spin-coated **β,β′-C**_***n***_**-TIFDMT** thin films were studied by atomic force microscopy (AFM) and out-of-plane grazing-incidence X-ray diffraction (GIXRD) techniques. The GIXRD technique at an incidence angle of 0.5° was preferred over a Bragg–Brentano geometry in order to reveal subtle microstructural differences between varied **β,β′-C**_***n***_**-TIFDMT** thin films. As shown in Figure 5, all deposited molecular thin films showed small (∼50–100 nm) highly interconnected nodular-like grains in their 100 °C annealed samples. The crystallinities in this stage were found to be quite poor. Integration of these nodules into larger crystalline domains was observed to start at 120 °C annealing temperature, which yielded two-dimensional (sub)micrometer-sized, sharp-edged plate-like grains lying parallel with the substrate plane after annealing at 150 °C. Annealing at even higher temperatures had a minimal effect on these morphologies. While very large micrometer-sized (∼1–3 μm) grains were observed for 150 °C annealed **β,β′-C**_**12**_**-TIFDMT** and **β,β′-C**_**16**_**-TIFDMT** thin films, a similarly prepared **β,β′-C**_**8**_**-TIFDMT** thin film showed relatively smaller grains (∼300–400 nm). A sufficient alkyl chain length (*n* ≥ 12) seems to be a key factor herein to achieve micrometer-sized crystalline domains.

**Figure 6:**
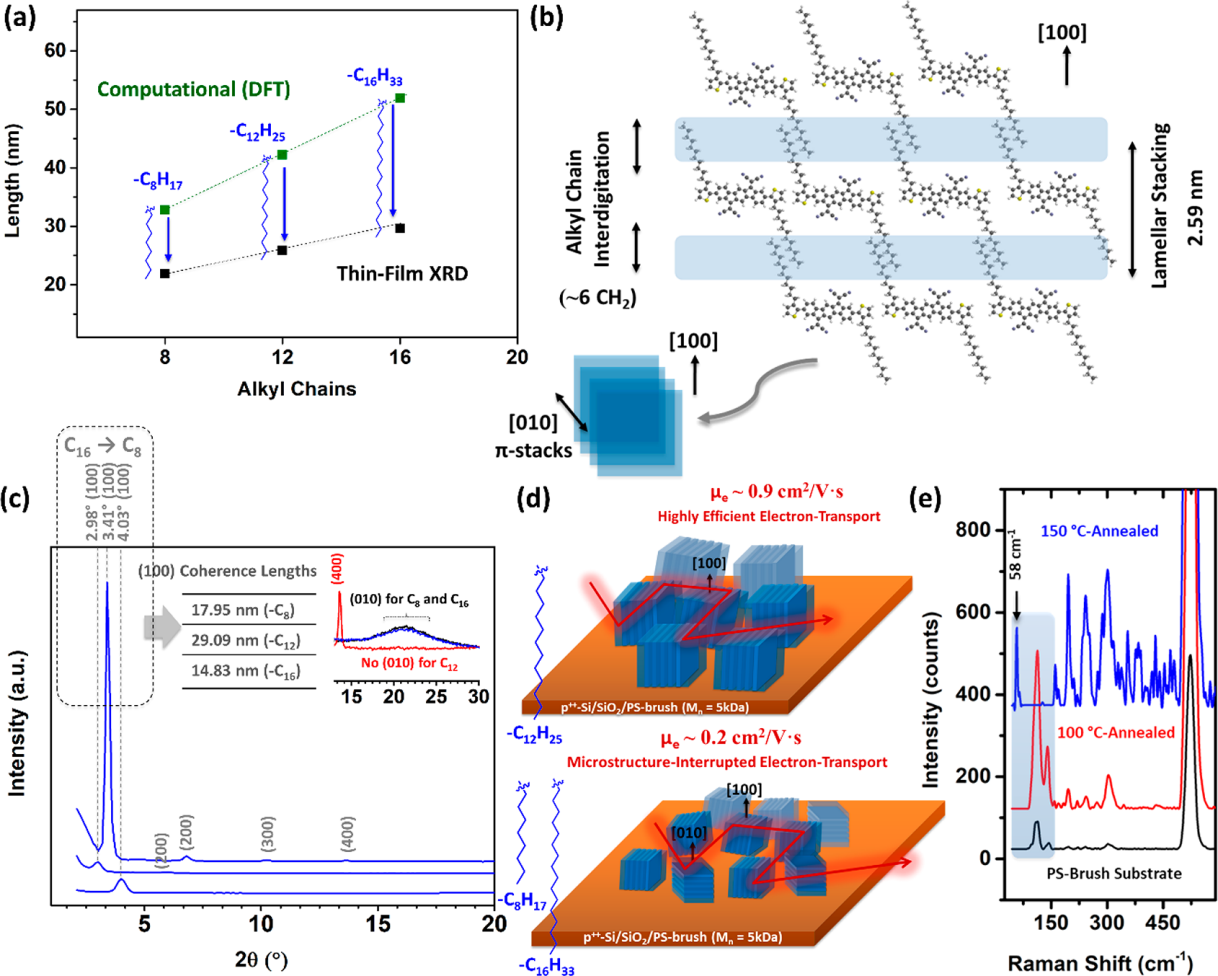
(a) Experimental *d* spacings (black squares) for 150 °C annealed thin films and computationally optimized **β,β′-C**_***n***_**-TIFDMT** molecular lengths with fully extended all-trans alkyl chains (green squares) versus the number of carbon atoms in the substituents. The difference for each alkyl chain length is shown with a blue arrow. (b) Schematic model of the **β,β′-C**_***n***_**-TIFDMT** thin film lamellar stacking with interdigitated alkyl chains (the example shown is for C_12_) and the corresponding crystallographic [100] and [010] π-stack directions. (c) Out-of-plane grazing-incidence X-ray diffraction (GIXRD) pattern (θ_incident_ = 0.5°) of the **β,β′-C**_***n***_**-TIFDMT** thin films on p^++^-Si/SiO_2_/PS-brush (*M*_n_ = 5 kDa) annealed at 150 °C illustrating the differences in the (100) and (010) diffraction peaks for C_8_, C_12_, and C_16_. The crystallite size for each thin film is estimated using the Scherrer equation. (d) Schematic of the crystallite domain orientations of the **β,β′-C**_***n***_**-TIFDMT** thin films (C_12_ (top) vs C_8_ and C_16_ (bottom)) on the p^+2^-Si/SiO_2_/PS-brush (*M*_n_ = 5 kDa) substrates. (e) Low-frequency Raman spectra of p^++^-Si/SiO_2_/PS-brush (*M*_n_ = 5 kDa) substrates (black line) and the **β,β′-C**_**12**_**-TIFDMT** thin film on p^++^-Si/SiO_2_/PS-brush (*M*_n_ = 5 kDa) after 100 °C (red line) and 150 °C thermal annealing (blue line) (for 30 min) under vacuum.

The XRD profiles of the present **β,β′-C**_***n***_**-TIFDMT** thin-films exhibit one low-angle (2θ = 3.60–4.03° for −C_8_, 2.73–3.41° for −C_12_, and 2.84–2.98° for −C_16_) (100) diffraction peak for each molecule (Figure 5 insets). When the annealing temperature increases as 100 → 120 → 150 °C, the intensity of the (100) peak increases, the full-width at half-maximum (fwhm) value decreases, and higher order diffractions of the same family (up to (400) for −C_12_ and (200) for −C_16_) develop. All these changes point to a thermally induced thin-film crystallization behavior on the low-energy PS-brush interlayer, which is consistent with the aforementioned morphological improvements. The *d* spacings for the 150 °C annealed thin films were calculated to be 21.90 Å for **β,β′-C**_**8**_**-TIFDMT**, 25.88 Å for **β,β′-C**_**12**_**-TIFDMT**, and 29.61 Å for **β,β′-C**_**16**_**-TIFDMT**. Upon comparing *d* spacings with the geometry-optimized, computed molecular dimensions of **β,β′-C**_***n***_**-TIFDMT** (Figure S28), we see that although they are longer than the TIFDMT π-length of 20.51 Å, they can be related to the molecular lengths perpendicular to the long π-axis. Moreover, the *d* spacings gradually increase with the alkyl chain length (*n* = 8 → 12 → 16). These indicate that the present thin films have a long-range ordered edge-on lamellar packing with alternating σ- and π-layers, and the alkyl substituents extend along the out-of-plane direction as illustrated in Figure 6b.^53^ It is worth noting that the high μ_e_ values observed in the **β,β′-C**_***n***_**-TIFDMT** thin films provide additional evidence for edge-on lamellar packing. This is because such packing allows for strong interlamellar π-interactions along the substrate surface, facilitating an efficient source-to-drain electron transport.^15,51,54^ As evidenced by optical characterizations (*vide supra*), the π-interactions appear to be in a J-aggregate-like slipped-stacked arrangement, which could also maximize the utilization of these dispersive interactions between alkyl chains of the adjacent lamellae. Figure 6a illustrates that when plotting the experimental *d* spacings of the semiconductor against the number of carbon atoms in its alkyl substituent, the resulting slope was determined to be 0.96 Å per methylene (−CH_2_−) unit. This value is lower than the computed increase in all-trans alkyl substituent length (1.24 Å/methylene). The difference points to a tilt angle of ∼39° for the alkyl substituents from the substrate surface normal, which matches well with the geometry-optimized, computed molecular structures having all-trans alkyl substituents at an angle of ∼37–42° with respect to the long molecular axis (Figure S28). The observed *d* spacings for −C_8_ (24.51 Å) and −C_12_ (32.32 Å) at 100 °C annealing temperature compare well with the maximum possible computed molecular lengths (24.34 and 32.07 Å, respectively, Figure S28) with all-trans alkyl substituents. This also suggests that alkyl chains are not interdigitating at 100 °C annealed thin films.

As shown in Figure 5 insets, the (100) peak for each semiconductor was found to shift to a larger 2θ value upon thermal annealing, and the corresponding *d* spacing successively decreases as 24.51 Å → 21.90 Å → 21.90 Å for **β,β′-C**_**8**_**-TIFDMT**, 32.32 Å → 31.52 Å → 25.88 Å for **β,β′-C**_**12**_**-TIFDMT**, and 31.07 Å → 30.22 Å → 29.61 Å for **β,β′-C**_**16**_**-TIFDMT**. In view of the fact that the *d* spacing shortenings are accompanied by a strong thin-film crystallization and an increase in OFET electron mobilities, alkyl chain interdigitation is undoubtedly the key element. This is because a simple molecular tilt on the surface, which could be the only other option for the *d* spacing shortening, is highly unlikely to promote a large crystallization to yield micrometer-sized grains. Also, a molecular tilt may impede electron mobility, as the alkyl chains could adopt in-plane conformations and become more likely to disrupt source-to-drain charge transport.^55,56^ On the other hand, dispersive interactions that could form over a large number of methylene units between flexible alkyl chains could induce a strong crystallization behavior via the so-called “fastener” (or “zipper”) effect.^39,40,57^ These interactions not only occur within the lamellae to increase crystallite size in the out-of-plane direction but also promote in-plane crystal growth as evidenced in the AFM morphologies. We note that the cohesive energetics for an interdigitation of six methylene units, as we observed in the case of **β,β′-C**_**12**_**-TIFDMT**, could promote a large crystallization energy of ∼50–60 kJ mol^–1^ of a molecule (8.9 ± 0.6 kJ mol^–1^ per each methylene unit),^58,59^ which can rearrange the in-plane π-interactions over large domains. Moreover, this type of interdigitation effectively maintains the insulating σ-alkyl chains within the lamellar plane, preventing them from hindering the charge transport between the source and drain. The degree of interdigitation was found to increase with the alkyl chain length; while final interdigitations of ∼6 and ∼10 methylene units were estimated for **β,β′-C**_**12**_**-TIFDMT** and **β,β′-C**_**16**_**-TIFDMT**, respectively, it was found to be more limited for **β,β′-C**_**8**_**-TIFDMT** involving ∼2 methylene units. Interestingly, the interdigitation for all semiconductors seems to propagate in the σ-layer of the lamellae until the first six methylene units (−(CH_2_)_6_–TIFDMT–(CH_2_)_6_−) are attached to the π-system. At this point, our attention is directed toward the alteration in the degree of interdigitation upon thermal annealing, rather than the ultimate magnitude of interdigitation. This alteration should, indeed, be the pivotal mechanism driving the process of thermally induced thin-film crystallization. Among the current thin films, the largest decrease in *d* spacing occurred for **β,β′-C**_**12**_**-TIFDMT**. While the *d* spacing shortening for −C_8_ and −C_16_ is only ∼1.5–2.5 Å, −C_12_ shows a larger effect with ∼6.5 Å, indicating that a larger number of methylene units are interdigitating (enhanced cohesive energetics)^33,40,60^ to drive the crystallization process. This effect was also apparent in the (100) coherence lengths (*L*) (i.e., extent of ordering in the out-of-plane direction), which were estimated using the Scherrer equation^48,61^
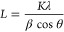
3in which *K* is the Scherrer constant (0.9), λ is the wavelength of the radiation for the Cu Kα X-ray source (0.15406 nm), and β and θ are the fwhm and the peak position values for the diffraction peaks, respectively, in radians. As shown in Figure 6c, a large (100) coherence length of 29.09 nm was calculated for −C_12_ when all (100)–(400) diffractions peaks were taken into consideration, whereas −C_8_ and −C_16_ showed relatively smaller lengths of 17.95 and 14.83 nm, respectively (see Figure S29 for Gaussian fittings). In addition, higher order diffraction peaks up to the fourth order appear only for **β,β′-C**_**12**_**-TIFDMT** thin films, confirming the presence of a long-range crystal ordering.

Finally, a subtle difference was evident in the GIXRDs. As shown in Figure 5, **β,β′-C**_**8**_**-TIFDMT** and **C**_**16**_**-TIFDMT** thin films show a broad peak at 2θ ≈ 20–21° at all annealing temperatures, which is assigned to short-range interlamellar π-interactions (∼4.1 Å) between (010) planes. The same (010) peak was also evident for **β,β′-C**_**12**_**-TIFDMT** at 100 and 120 °C annealed thin films. However, this peak completely disappears for the **β,β′-C**_**12**_**-TIFDMT** thin film after 150 °C annealing (Figure 6c inset). This indicates the presence of only (100) planes in the out-of-plane direction after complete crystallization without any unfavorable (010) planes (Figure 6d). The effect of edge-on vs face-on crystallite ratios on OFETs was recently demonstrated for the polycrystalline thin film of an ambient-stable n-type semiconductor, and 2–4-fold decreases were reported with increased (010)-oriented crystallite ratio.^62^ On this basis of these findings, the C_12_ alkyl chains seem to induce the optimal cohesive energetics for the formation of large crystallites without (010) interruptions on the PS-brush interlayer. While going from −C_8_ to −C_12_ is apparently an effective strategy for small molecules to realize additional crystallization via alkyl chain interdigitation, much longer −C_16_ chains seem to induce extended alkyl chain interactions even at lower annealing temperatures without leaving additional room for further interdigitation and, thus, crystallization. On the other hand, it is noteworthy that the annealing temperature of 150 °C employed for the present **β,β′-C**_***n***_**-TIFDMT** semiconductors, which yielded the best semiconductor thin-film morphology/microstructure and the highest electron mobility, is above the reported glass transitions of low-molecular-weight polystyrene-based thin films^63^ and brushes.^64^ One could expect a deteriorating effect on charge transport efficiency due to interfacial viscoelasticity of the polymer interlayer at elevated temperatures.^22,63,65^ In contrast, the increased μ_e_ values (>100–1000×) measured in our case clearly suggests that the thermally induced, strong crystallization behavior in the already existing, top-lying semiconductor layer (∼40–45 nm thickness vs 3.6–6.6 nm for polymer interlayer) minimizes the interfacial viscoelasticity effect and governs the microstructural and morphological arrangement, as evidenced in our characterizations (*vide supra*). Note that, for the previously reported PS-brush interlayers, the semiconductor layer was slowly deposited via thermal evaporation, which allows for the polymer interlayer viscoelasticity to interfere with the semiconductor crystallization during the early stages of film growth.^63,65^ The excellent crystallinity for **β,β′-C**_**12**_**-TIFDMT** on the PS-brush interlayer even allowed us to observe external crystal lattice vibrations (phonons) of the present organic thin film by using low-frequency Raman (<200 cm^–1^) scattering. Due to the large molecular weight and weakness of intermolecular forces, these lattice vibrations typically occur at low frequencies for organic crystals.^66,67^ As shown in Figure 6e, the **β,β′-C**_**12**_**-TIFDMT** thin film, after annealing at 150 °C, exhibited a clear, high-intensity peak at 58 cm^–1^ while the underlying substrate-based signal became suppressed (see Figure S30 for the full spectra). Note that this low-frequency peak did not exist in the 100 °C annealed semiconductor thin film, as well as in the p^++^-Si/SiO_2_/PS-brush substrate. On the other hand, when the current **β,β′-C**_***n***_**-TIFDMT** semiconductor thin films were fabricated on the *M*_n_ = 22 kDa PS-brush (Figure S31), while lower average electron mobilities of 0.06–0.11 cm^2^/(V·s) were measured at an annealing temperature of 150 °C for −C_8_ and −C_16_, **β,β′-C**_**12**_**-TIFDMT** thin films maintained their high average electron mobility of 0.48 cm^2^/(V·s) (μ_e_^max^ = 0.81 cm^2^/(V·s)). This clearly suggests that a relatively lower grafting density of PS-22 (0.19 chains/nm^2^ vs 0.45 chains/nm^2^ of PS-5) still offers a highly favorable polymer interlayer surface for a high-mobility n-type semiconductor, whereas it somewhat affects the charge-transport characteristics (Δμ_e_ ≈ 3–4×) for **β,β′-C**_**8**_**-TIFDMT** and **β,β′-C**_**16**_**-TIFDMT**. The AFM and XRD characterizations on PS-22 (Figure S31e,h) compare well with those on PS-5, explaining the observed high electron mobility for the **β,β′-C**_**12**_**-TIFDMT** thin film even on the PS-22 interlayer surface. Finally, we note that the output curves (Figure S27) exhibited a certain level of contact resistance, possibly arising from an energetic mismatch between the work function of the Au electrode and the unoccupied transport states of **β,β′-C**_***n***_**-TIFDMT**s or due to electronic/morphological interfacial effects between the organic semiconductor and the metallic electrode.^68,69^ Future investigations that explore alternative metallic/organic source-drain contacts, utilize self-assembled monolayers on the electrode surface, and incorporate charge injection layers or contact dopants have the potential to reduce contact resistance and even further enhance electron mobility.^70^

## Conclusions

In summary, a library of solution-processable, low-LUMO (−4.20 eV) 2,2′-(2,8-bis(3-alkylthiophen-2-yl)indeno[1,2-*b*]fluorene-6,12-diylidene)dimalononitrile small molecules, **β,β′-C**_***n***_**-TIFDMTs**, having varied alkyl chain lengths (*n* = 8, 12, 16) has been designed and synthesized. The physicochemical and optoelectronic properties of the new molecules have been studied in detail. The decreased solubility as the alkyl chain length increases was found to correlate well with the increased solid–isotropic liquid transition enthalpies. This correlation strongly indicates that cohesive energetics, tuned via alkyl chains, play a pivotal role in determining solubility. Moreover, it has been observed that all molecules undergo partial thermolysis of the terminal alkylthienyl end units at temperatures of 500–600 °C. The HOMO and LUMO energies were estimated as −5.75 and −4.20 eV, respectively. The semiconductor thin films were prepared under ambient conditions via spin-coating on densely packed, ultrathin PS-brush surfaces. Going from solution to the solid-state (after annealing at 150 °C), while optical characterizations suggested J-aggregate formation in thin films, morphological characterizations exhibited two-dimensional, micrometer-sized (∼1–3 μm), sharp-edged plate-like grains lying parallel with the substrate plane. OFETs fabricated by the current **β,β′-C**_***n***_**-TIFDMT** molecules showed excellent n-channel behavior under ambient with μ_e_ values reaching ∼0.9 cm^2^/(V·s), *I*_on_/*I*_off_ ≈ 10^7^–10^8^, and *V*_on_ ≈ 0 V. Detailed microstructural and morphological characterizations have given us key insights into the present thin-film crystallization mechanisms. Our findings revealed that thermally induced dispersive interactions occurring over a large number of methylene units between flexible alkyl chains (i.e., zipper effect) are critical to achieve a favorable thin-film crystallization on the current PS-brush interlayer. While going from −C_8_ to −C_12_ is apparently an effective strategy for small molecules to realize additional crystallization via alkyl chain interdigitation, much longer −C_16_ chains seem to induce extended alkyl chain interactions even at lower annealing temperatures without leaving additional room for further interdigitation and, thus, crystallization. Therefore, the optimal zipper effect was evident with C_12_ chains, which resulted in the formation of large crystallites having lamellar stacking ((100) coherence length ∼30 nm) in the out-of-plane direction with no face-on (010)-oriented crystallites. This particular microstructure could induce very efficient in-plane S → D charge transport and lies at the origin of the impressive electron mobility we have observed with the current **β,β′-C**_**12**_**-TIFDMT**-based OFETs. The excellent crystallinity of the **β,β′-C**_**12**_**-TIFDMT** thin film was also evident in the observed crystal lattice vibrations (phonons) at 58 cm^–1^ in low-frequency Raman scattering. Our study, by combining all three key transistor characteristics (μ_e_, *I*_on_/*I*_off_, and *V*_on_), not only demonstrates one of the finest solution-processed n-channel OFET devices reported under ambient conditions but also highlights the significant potential of electron-deficient indenofluorene π-cores for the development of high-performance semiconductors. In a broader context, the utilization of donor–acceptor type π-systems with alkyl substituents positioned along the short molecular axis exhibits great potential as high-performance semiconductors in future research endeavors. This architecture possesses a natural void space created by the alkyl substituents, allowing for interdigitation of alkyl chains. By carefully tuning the length of the alkyl substituents and the underlying surface properties, optimal chain interdigitation and cohesive energetics can be achieved, leading to favorable microstructure and morphology for highly efficient electron transport.

## Supporting Information Available

Syntheses and experimental protocols of intermediate compounds including ^1^H/^13^C NMR and mass spectra, chemical structures of previously developed ambient-stable and solution-processable n-type molecular semiconductors, proposed thermolysis reactions, DSC-based thermodynamic quantities, OFET output plots, computed molecular dimensions, Gauss fittings for diffraction peaks, Raman spectra of thin films, OFETs and the corresponding morphological/microstructural characterizations on PS-Brush (*M*_n_ = 22 kDa) interlayers (PDF)
