# Supplementary material for: Solution-Processable Indenofluorenes on Polymer Brush Interlayer: Remarkable N-Channel Field-Effect Transistor Characteristics under Ambient Conditions
Source: ACS Appl Mater Interfaces. 2023 Aug 15;15(35):41666–79. doi: 10.1021/acsami.3c07365 (PMC10485804; doi:10.1021/acsami.3c07365)
Supplement: Supplementary file 1 — am3c07365_si_001.pdf [file am3c07365_si_001.pdf]

Supporting Information

Solution-Processable Indenofluorenes on Polymer Brush Interlayer: Remarkable N-Channel Field-Effect Transistor Characteristics in Ambient

Ayse Can[1], Ibrahim Deneme[1], Gokhan Demirel[2]*, Hakan Usta[1*]

[1] Department of Nanotechnology Engineering, Abdullah Gül University, 38080 Kayseri, Turkey.
[2] Bio-inspired Materials Research Laboratory (BIMREL), Department of Chemistry, Gazi University, 06500 Ankara, Turkey.

*Address correspondence to:
Prof. Hakan Usta (hakan.usta@agu.edu.tr)
Prof. Gokhan Demirel (E-mail: nanobiotechnology@gmail.com)

Table S1. The molecular structures, LUMO energetics, OFET device configurations/performances, and semiconductor thin-film deposition methods of previously reported *n*-type ambient-stable molecular semiconductors with high electron mobilities ($\mu_e \geq 0.5$-$1.0\ cm^2/V\cdot s$) and current modulation characteristics ($I_{on}/I_{off} \geq 10^6$-10^7).

Molecular Structure	LUMO (eV)	OFET Performance I_{on}/I_{off}, μ_e ($cm^2/V\cdot s$), V_{th} (V)	Thin-film Deposition Method	Device Configuration	Reference
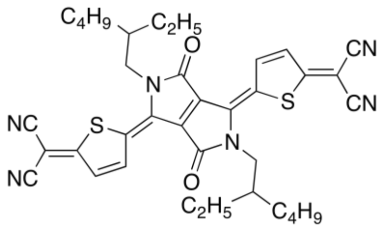	−4.51	10^6, 0.55, 9.5	Vapor deposition	Si/SiO_2/OTS/1/Au	Zhu et al.[1]
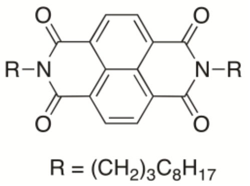	−3.71	10^5-10^6, 0.70, 3-13	Vapor deposition	Si/SiO_2/OTS/2/Au	Katz et al.[2]
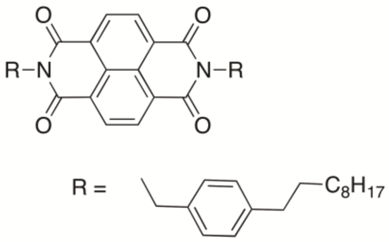	-	10^7, 0.57, 13-48	Vapor deposition	Si/SiO_2/OTS/3/Au	Katz et al.[3]

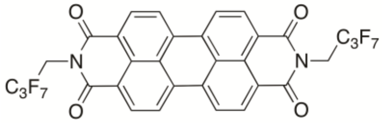	−3.85	10^6, 1.24, 47-57	Vapor deposition	Si/SiO_2/OTS/4/Au	Würthner et al.[4]
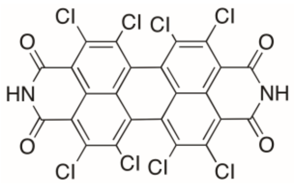	−4.23	10^8, 0.82, 28	Vapor deposition	Si/SiO_2/OTS/5/Au	Würthner et al.[5]
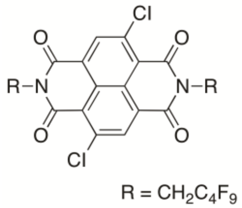	−4.01	10^7, 1.43, 23±2	Vapor deposition	Si/SiO_2/OTS/6/Au	Bao et al.[6]
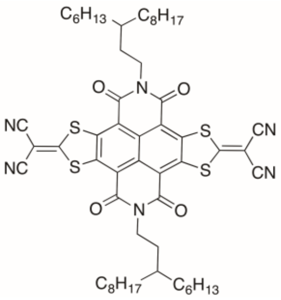	−4.38	10^8, 3.50, 23±2	Solution deposition	Si/SiO_2/OTS/Au/7	Zhu et al.[7]

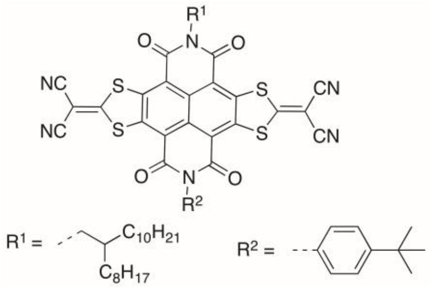	−4.32	10^7, 0.70, 7	Solution deposition	Si/SiO_2/OTS/8/Au	Zhu et al.[8]
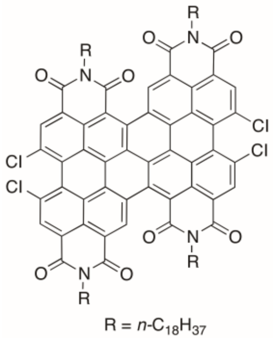	−4.30	10^7, 0.70, 21	Solution deposition	Si/SiO_2/OTS/9/Au	Wang et al.[9]
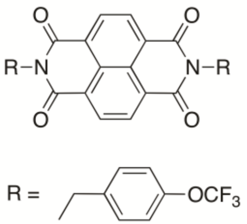	−4.22	10^6, 0.70, 21	Vapor deposition	Si/SiO_2/OTS/10/Au	Meng et al.[10]

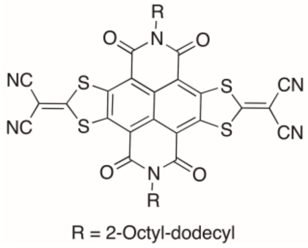	−4.3	10^6-10^7, 0.51, 21	Solution deposition	Si/SiO_2/OTS/11/Ag	Zhu et al.[11]
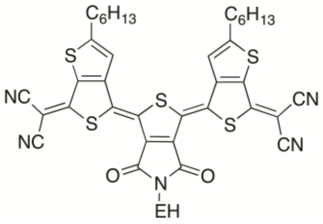	−4.51	10^6, 3.0, −1.4	Solution deposition	Si/SiO_2/OTS/Au/12	Zhu et al.[12]
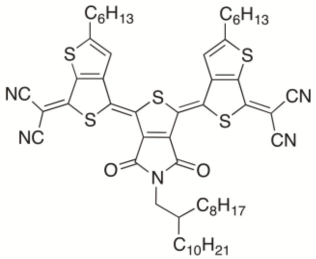	−4.44	10^6, 5.2, −13.9	Solution deposition	Si/SiO_2/OTS/Au/13	Zhu et al.[13]
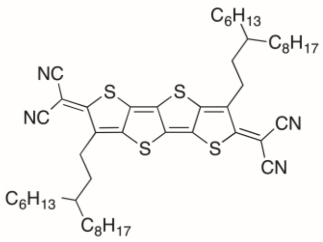	−4.3	10^6, 0.90, -	Solution deposition	Si/SiO_2/OTS/14/Au	Zhu et al.[14]

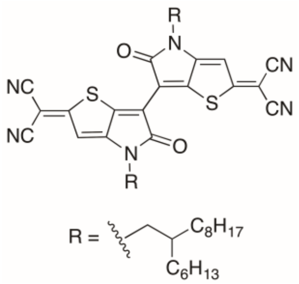	−4.16	10^5-10^6, 2.54, 16.2±7.23	Solution deposition	Si/SiO_2/PETS/15/Ag	Facchetti et al.[15]

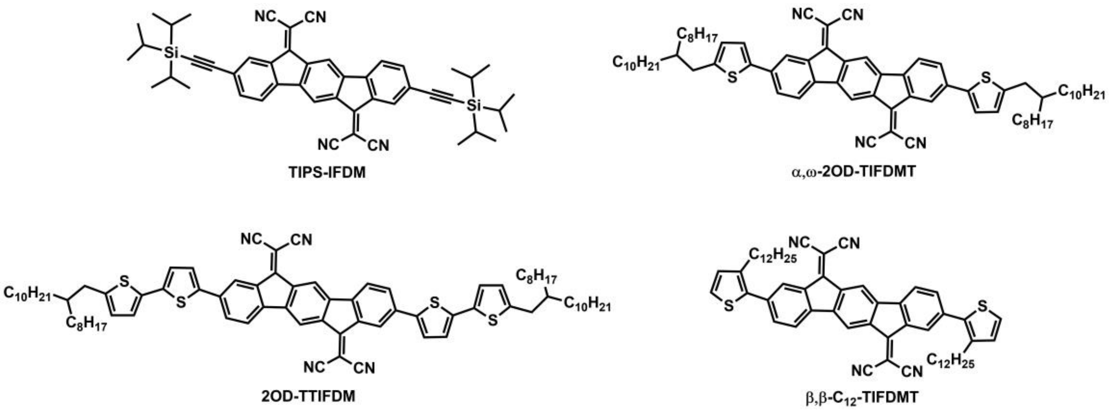

Figure S1. The chemical structures of our previously designed and synthesized ambient-stable *n*-type **IFDM**-based molecular semiconductors, **TIPS-IFDM**,[16] **α,ω-2OD-TIFDMT**,[17] **2OD-TTIFDM**,[18] and **β,β'-C$_{12}$-TIFDMT**[19].

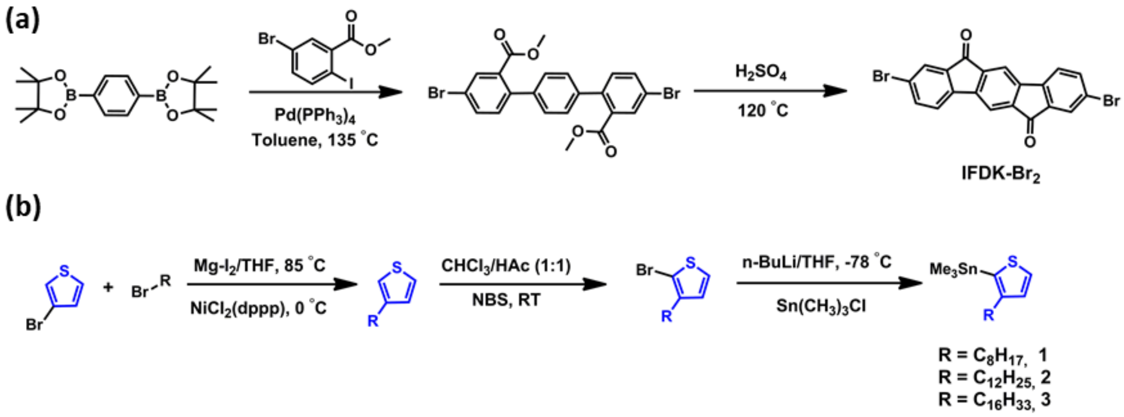

Scheme S1. The synthesis of **IFDK-Br$_2$** (a) and (3-alkylthiophen-2-yl)trimethylstannane reagents **1-3** (b).

Synthesis of 4,4''-dibromo-2,2''-methoxycarbonyl-[1,1';4',1'']terphenyl: A mixture of 1,4-benzenediboronic acid bis(pinacol) ester (1.70 g, 5.15 mmol), methyl 2-iodo-5-bromobenzoate (3.85 g, 11.28 mmol), and Aliquat 336 (0.641 mL) was dissolved with anhydrous toluene (35 ml) under nitrogen. Then, tetrakis(triphenylphosphine)palladium ($Pd(PPh_3)_4$) (0.36 g, 0.31 mmol) and 1M aqueous sodium carbonate (Na_2CO_3) solution (2.23 g in 21.0 mL of distilled water) were added under nitrogen. The reaction mixture was stirred at 135 °C for 48 hours. The resulting reaction mixture was allowed to cool down to room temperature. The reaction mixture was quenched with water and extracted with hexanes. The organic phase was washed with water, dried over Na_2SO_4, filtered, and evaporated to dryness to give a crude product, which was purified by column chromatography on silica gel using chloroform as the eluent. Pure product was afforded as a white solid (2.17 g, 84% yield). ^1H NMR ($CDCl_3$): δ 8.00 (s, 2H), 7.68 (d, 2H, J = 8.0 Hz), 7.33 (s, 4H), 7.31(d, 2H, J = 4.0 Hz), 3.71 (s, 6H) ppm.

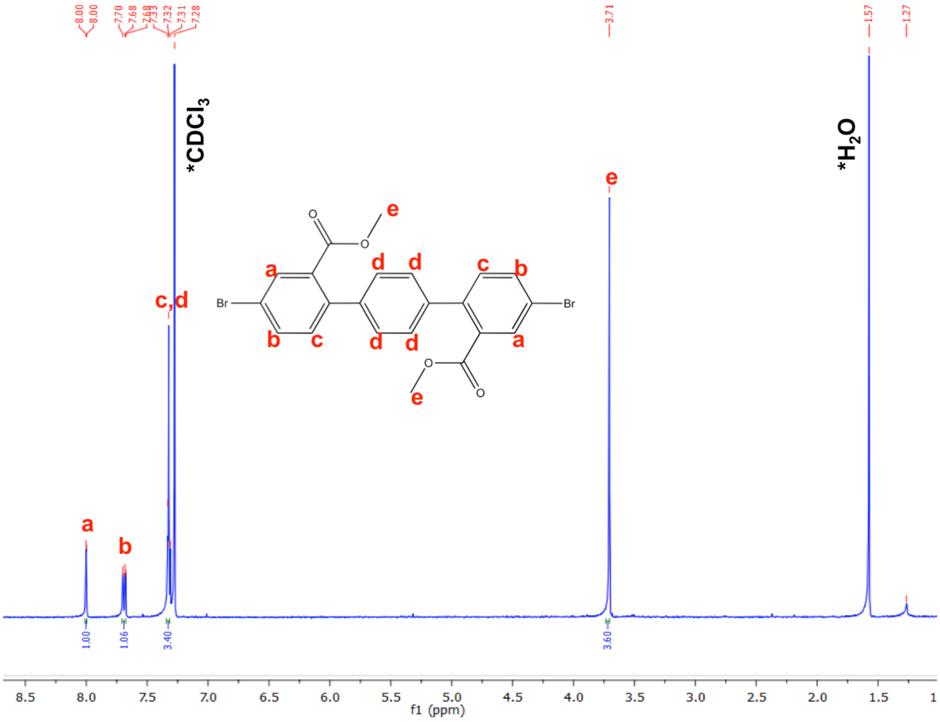

Figure S2. ^{1}H NMR spectrum of **4,4’’-dibromo-2,2’’-methoxycarbonyl-[1,1’;4’,1’’]terphenyl** in $CDCl_3$ at room temperature. $CDCl_3$ and H_2O peaks in NMR solvent are denoted by asterisks.

Synthesis of 2,8-dibromo-indeno[1,2-b]fluorene-6,12-dione (IFDK-Br_2): The diester 4,4’’-dibromo-2,2’’-methoxycarbonyl-[1,1’;4’,1’’]terphenyl (0.55 g, 1.09 mmol) was added to 50.0 mL of 80 % H_2SO_4 (prepared from 10.0 mL of H_2O and 40.0 mL of concentrated (99.99 %) H_2SO_4), and the mixture was stirred at 120 °C overnight, during which time the white solid turn ed dark red. The reaction mixture was next poured into ice and filtered to collect the red cryst als. The collected product was washed with concentrated sodium hydrogen carbonate ($NaHCO_3$) solution and filtered again. Then, the collected product was washed with methanol and filt ered to collect the crude product. The crude product was used for the next step without any fur ther purification. The product was afforded as a dark red solid (0.48 g, 74% yield). m.p: > 300 °C. MS (APCI) *m/z* (M^+) calcd. for $C_{20}H_8O_2Br_2$: 440.09, found: 440.5. Anal. calcd. for $C_{20}H_8O_2Br_2$: C, 54.58; H, 1.83 Found: C, 54.12; H, 1.72.

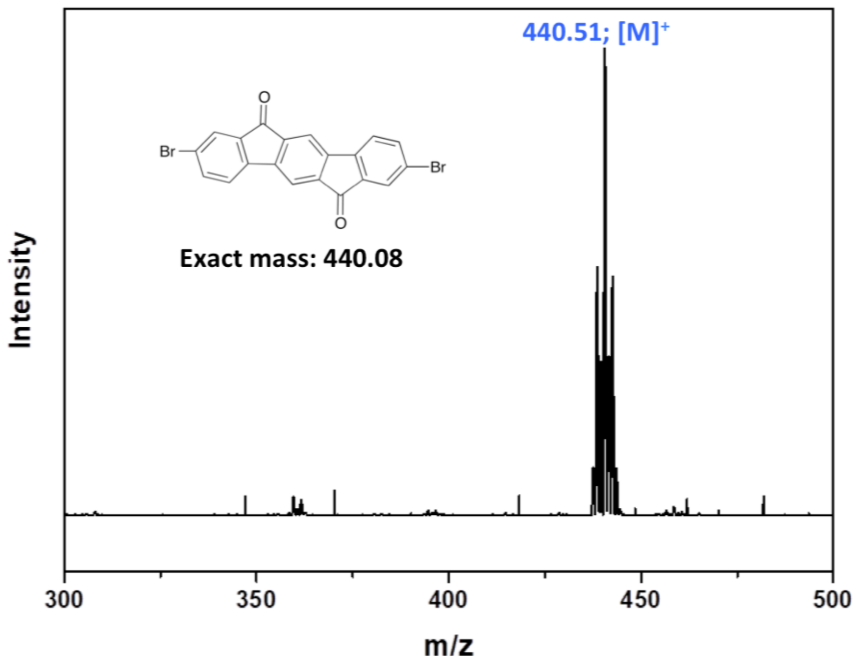

Figure S3. Positive ion MS-APCI spectrum of **IFDK-Br$_2$**.

Synthesis of 3-octylthiophene: To a solution of 1-bromooctane (2.95 mL, 16.99 mmol) in anhydrous THF (32.0 mL) magnesium (0.49 g, 20.08 mmol) and iodine (0.12 g, 0.46 mmol) were added under nitrogen and this mixture was stirred at 85 °C for 3 hours. The resulting Grignard reagent was allowed to cool down to room temperature. Then, the Grignard reagent was added slowly to a solution of 3-bromothiophene (1.45 mL, 15.45 mmol) and NiCl$_2$(dppp) (0.12 g, 0.23 mmol)in anhydrous THF (12.0 mL) at 0 °C under nitrogen. The reaction mixture was allowed to warm to room temperature overnight. The resulting reaction mixture was quenched with water and extracted with dichloromethane. The organic phase was washed with water, dried over Na$_2$SO$_4$, filtered, and evaporated to dryness to give a crude product, which was purified by column chromatography on silica gel using hexanes as the eluent. Pure product was afforded as colorless oil (2.31 g, 76% yield). ^{1}H NMR (CDCl$_3$): δ 7.25 (s, 1H), 6.94 (d, 2H, J = 8.0 Hz), 2.62 (t, 2H, J = 12.0 Hz), 1.28 (d, 12H, J = 16.0 Hz), 0.88 (t, 3H, J = 12.0 Hz) ppm.

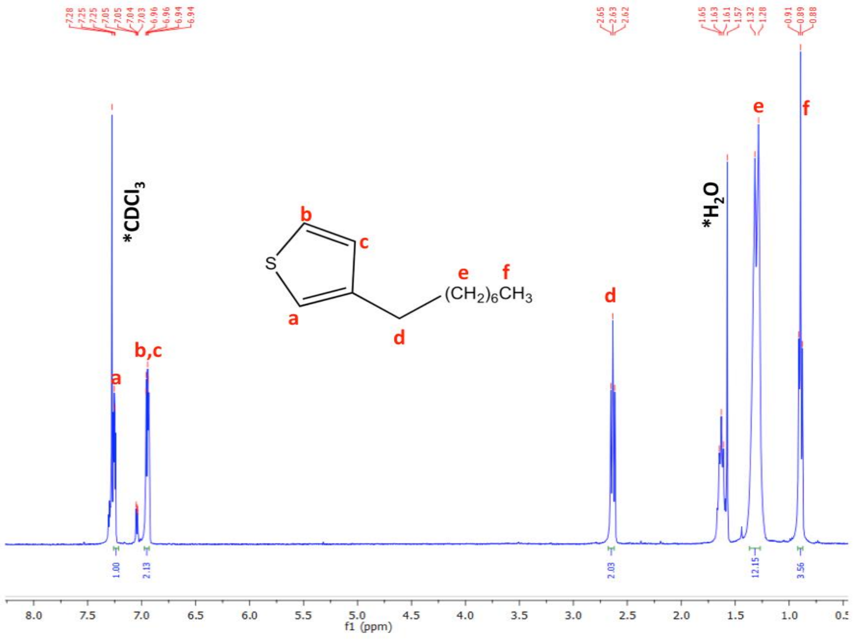

Figure S4. ^{1}H NMR spectrum of **3-octylthiophene** in $CDCl_3$ at room temperature. $CDCl_3$ and H_2O peaks in NMR solvent are denoted by asterisks.

Synthesis of 2-bromo-3-octylthiophene: To a solution of 3-octylthiophene (2.31 g, 11.74 mmol) in chloroform (50 mL): acetic acid (50 mL) (1:1 (v/v)) N-bromosuccinimide (NBS) (2.20 g, 12.33 mmol) was added under nitrogen. The mixture was stirred at room temperature overnight. The resulting reaction mixture was quenched with water and extracted with dichloromethane. The organic phase was washed with water, dried over Na_2SO_4, filtered, and evaporated to dryness to give a crude product, which was purified by column chromatography on silica gel using hexanes as the eluent. Pure product was afforded as light yellow oil (1.94 g, 59% yield). ^{1}H NMR ($CDCl_3$): δ 7.19 (d, 1H, J = 4.0 Hz), 6.80 (d, 1H, J = 4.0 Hz), 2.55 (t, 2H, J = 16.0 Hz), 1.29 (d, 12H, J = 12.0 Hz), 0.88 (t, 3H, J = 12.0 Hz) ppm.

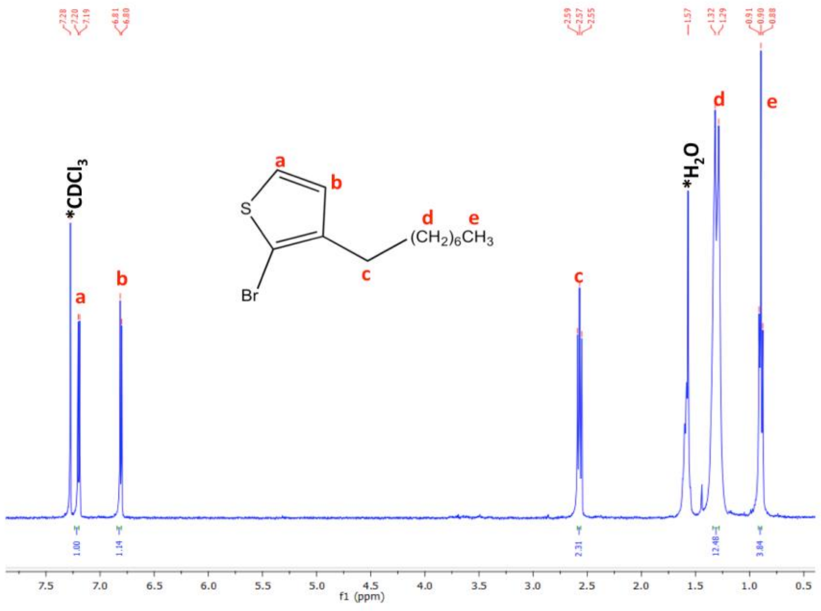

Figure S5. ^{1}H NMR spectrum of **2-bromo-3-octylthiophene** in $CDCl_3$ at room temperature. $CDCl_3$ and H_2O peaks in NMR solvent are denoted by asterisks.

Synthesis of 2-trimethyltin-3-octylthiophene (1): To a solution of 2-bromo-3-octylthiophene (1.94 g, 6.93 mmol) in anhydrous THF (50 ml) at -78 °C n-butyllithium (2.5 M in n-hexane) (2.91 ml, 7.28 mmol) was added dropwise under nitrogen. The mixture was stirred at −78 °C for 1 hour. Then, trimethyltinchloride ($Sn(CH_3)_3CI$) (1.52 g, 7.62 mmol)) was added at −78 °C, and the resulting reaction mixture was allowed to warm to room temperature overnight. The reaction mixture was quenched with water, and the product was extracted with hexanes. The organic phase was washed with water, dried over Na_2SO_4, filtered, and evaporated to dryness to give a crude product. The crude product was used for the next step without any further purification. The pure product was obtained as pale orange oil (2.41 g, 96% yield). ^{1}H NMR ($CDCl_3$): δ 7.54 (d, 1H, J = 4.0 Hz), 7.10 (d, 1H, J = 4.0 Hz), 2.61 (t, 2H, J = 16.0 Hz), 1.28 (d, 12H, J = 16.0 Hz), 0.88 (t, 3H, J = 12.0 Hz), 0.38 (s, 9H) ppm.

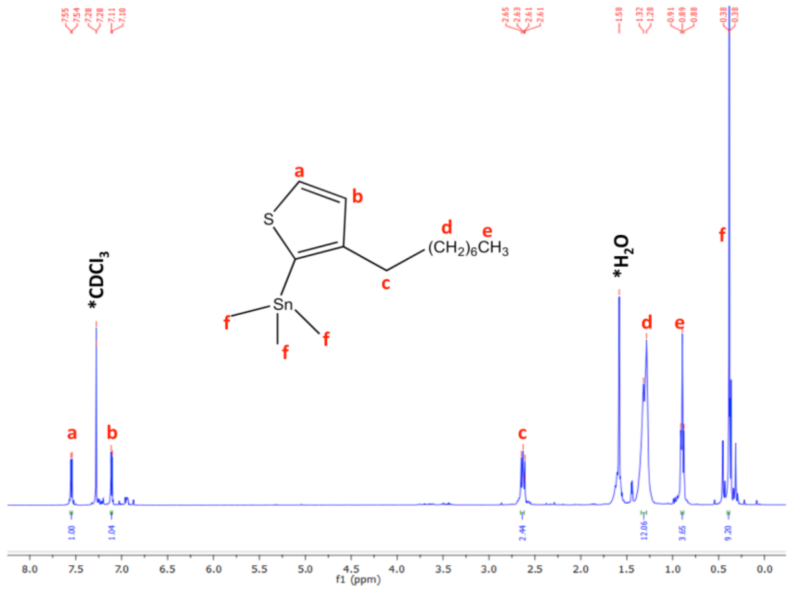

Figure S6. ^{1}H NMR spectrum of **1** in $CDCl_3$ at room temperature. $CDCl_3$ and H_2O peaks in NMR solvent are denoted by asterisks.

Synthesis of 2,8-di-3-octylthiophene-indeno[1,2-b]fluorene-6,12-dione (β,β'-C_8-TIFDKT): The mixture of 1 (0.628 g, 1.75 mmol), IFDK-Br_2 (0.350 g, 0.795 mmol), and $Pd(PPh_3)_2Cl_2$ (93.0 mg, 0.132 mmol) in anhydrous DMF (70.0 mL) were heated at 125 °C under nitrogen for 48 hours. The reaction mixture was cooled down to RT and evaporated to dryness. Then, the collected product was washed with methanol and filtered to collect the crude product, which was purified by column chromatography on silica gel using $CHCl_3$/hexanes (9:1 (v/v)) as the eluent. Finally, the product was washed with methanol and filtered to afford the final product as a purple solid (0.21 g, 40% yield). m.p.: 171-172 °C. ^1H NMR ($CDCl_3$): δ 7.86 (s, 2H), 7.78 (s, 2H), 7.63 (S, 4H), 7.29 (d, 2H, J = 4.0 Hz), 7.02 (d, 2H, J = 4.0 Hz) 2.67 (t, 4H, J = 16.0 Hz), 1.25 (t, 24H, J = 12.0 Hz), 0.85 (t, 6H, J = 12.0 Hz) ppm.

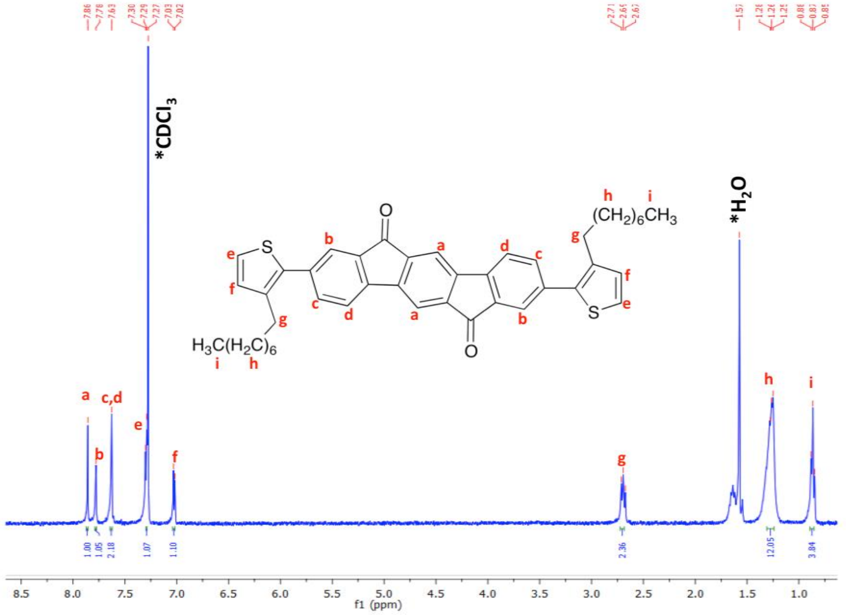

Figure S7. ^{1}H NMR spectrum of **2,8-di-3-octylthiophene-indeno[1,2-b]fluorene-6,12-dione (β,β'-C8-TIFDKT)** in $CDCl_3$ at room temperature. $CDCl_3$ and H_2O peaks in NMR solvent are denoted by asterisks.

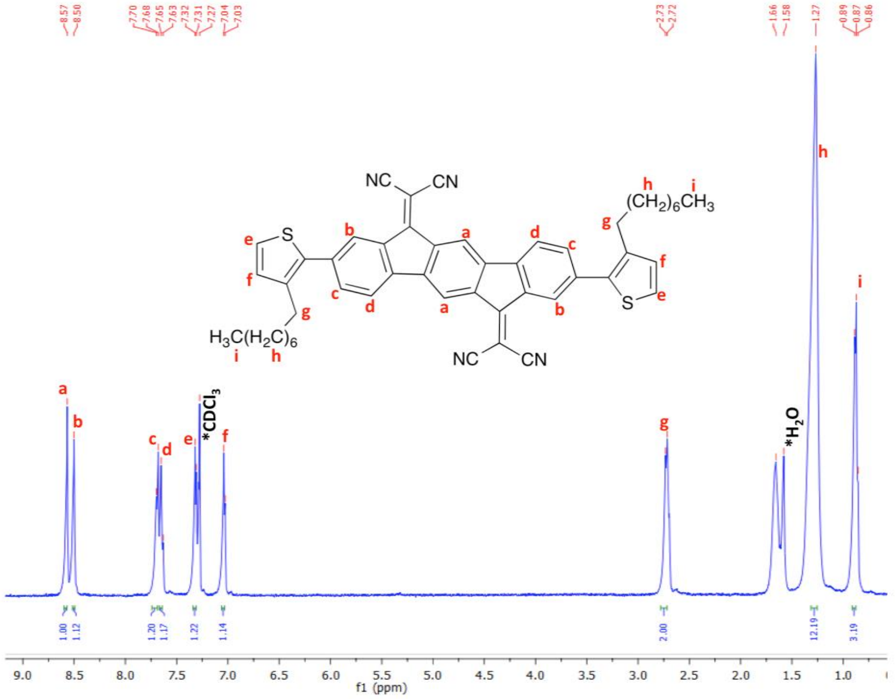

Figure S8. ^{1}H NMR spectrum of **2,8-di-3-octylthiophene-indeno[1,2-b]fluorene-6,12-dimalononitrile (β,β'-C8-TIFDMT)** in $CDCl_3$ at room temperature. $CDCl_3$ and H_2O peaks in NMR solvent are denoted by asterisks.

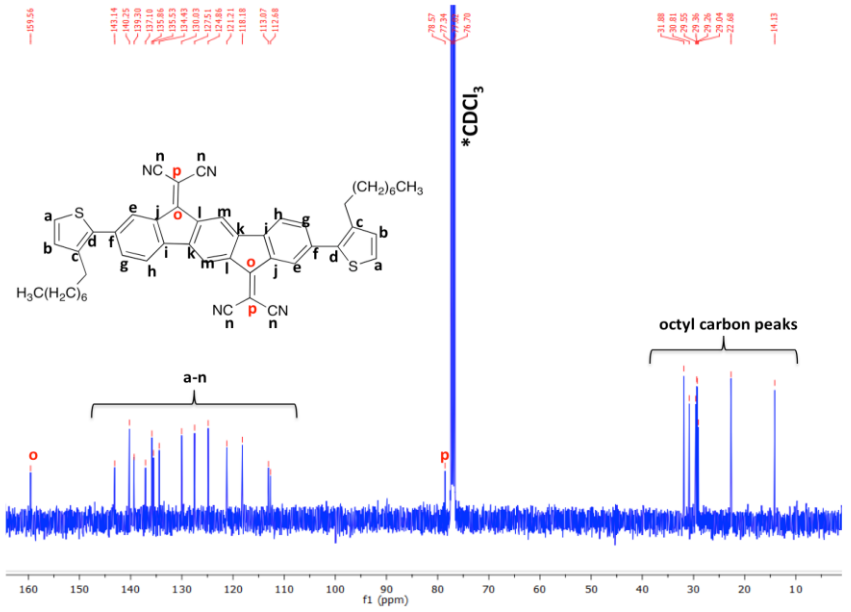

Figure S9. ^{13}C NMR spectrum of **2,8-di-3-octylthiophene-indeno[1,2-b]fluorene-6,12-dimalononitrile (β,β'-C_8-TIFDMT)** in $CDCl_3$ at room temperature. $CDCl_3$ peak in NMR solvent is denoted by an asterisk.

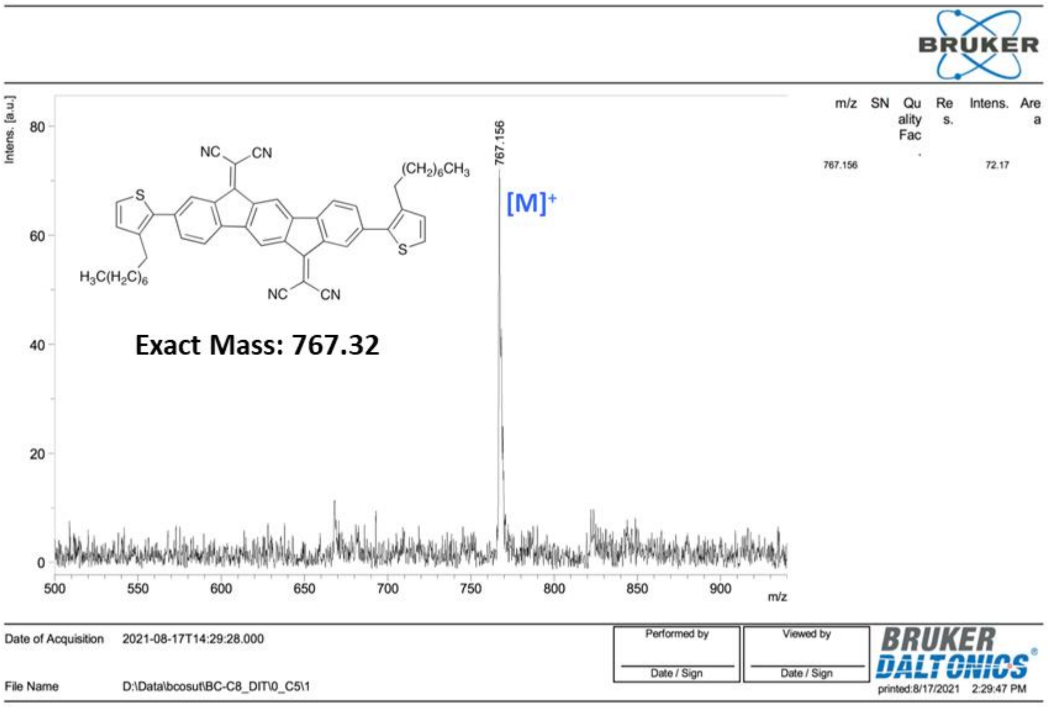

Figure S10. Positive ion and linear mode MALDI TOF-MS spectrum of **2,8-di-3-octylthiophene-indeno[1,2-b]fluorene-6,12-dimalononitrile (β,β'-C_8-TIFDMT)**.

Synthesis of 3-dodecylthiophene: To a solution of 1-bromooctane (4.08 mL, 16.99 mmol) in anhydrous THF (30.0 mL) magnesium (0.49 g, 20.08 mmol) and iodine (0.12 g, 0.46 mmol) were added under nitrogen and this mixture was stirred at 85 °C for 3 hours. The resulting Grignard reagent was allowed to cool down to room temperature. Then, the Grignard reagent was added slowly to a solution of 3-bromothiophene (1.45 mL, 15.45 mmol) and $NiCl_2$(dppp) (0.12 g, 0.23 mmol) in anhydrous THF (12.0 mL) at 0 °C under nitrogen. The reaction mixture was allowed to warm to room temperature overnight. The resulting reaction mixture was quenched with water and extracted with dichloromethane. The organic phase was washed with water, dried over Na_2SO_4, filtered, and evaporated to dryness to give a crude product, which was purified by column chromatography on silica gel using hexanes as the eluent. Pure product was afforded as colorless oil (2.80 g, 72% yield). ^1H NMR ($CDCl_3$): δ 7.29 (s, 1H), 6.95 (d, 2H, J = 12.0 Hz), 2.63 (t, 2H, J = 16.0 Hz), 1.29 (d, 20H), 0.90 (t, 3H, J = 4.0 Hz) ppm.

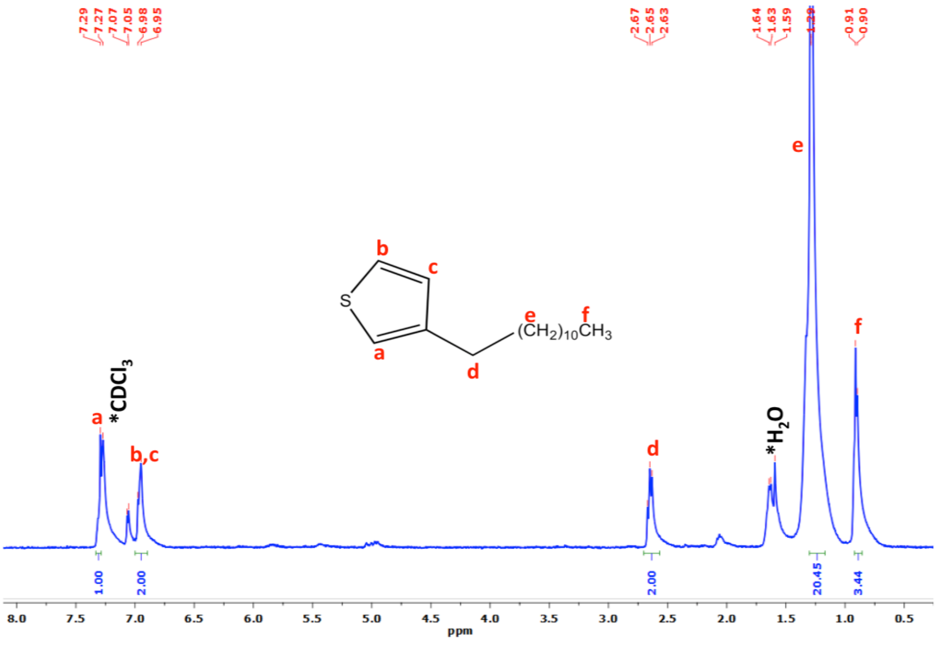

Figure S11. ^1H NMR spectrum of **3-dodecylthiophene** in $CDCl_3$ at room temperature. $CDCl_3$ and H_2O peaks in NMR solvent are denoted by asterisks.

Synthesis of 2-bromo-3-dodecylthiophene: To a solution of 3-dodecylthiophene (2.80 g, 11.12 mmol) in chloroform (50 mL): acetic acid (50 mL) (1:1 (v/v)) N-bromosuccinimide (NBS) (2.08 g, 11.68 mmol) was added under nitrogen. The mixture was stirred at room temperature overnight. The resulting reaction mixture was quenched with water and extracted with dichloromethane. The organic phase was washed with water, dried over Na_2SO_4, filtered, and evaporated to dryness to give a crude product, which was purified by column chromatography on silica gel using hexanes as the eluent. Pure product was afforded as light yellow oil (1.66 g, 45% yield). ^1H NMR ($CDCl_3$): δ 7.19 (d, 1H, *J* = 8.0 Hz), 6.80 (d, 1H, *J* = 8.0 Hz), 2.55 (t, 2H, *J* = 8.0 Hz), 1.27 (d, 20H, *J* = 16.0 Hz), 0.88 (t, 3H, *J* = 12.0 Hz) ppm.

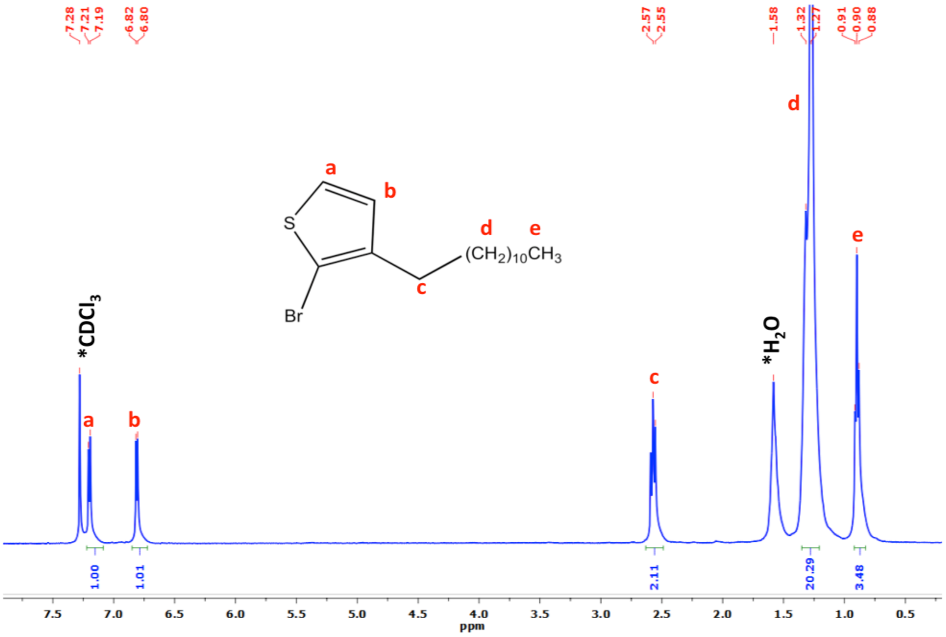

Figure S12. ^1H NMR spectrum of **2-bromo-3-dodecylthiophene** in $CDCl_3$ at room temperature. $CDCl_3$ and H_2O peaks in NMR solvent are denoted by asterisks.

Synthesis of 2-trimethyltin-3-dodecylthiophene (2): To a solution of 2-bromo-3-dodecylthiophene (1.66 g, 5.01 mmol) in anhydrous THF (45 ml) at -78 °C n-butyllithium (2.5 M in n-hexane) (2.10 ml, 5.26 mmol) was added dropwise under nitrogen. The mixture was stirred at −78 °C for 1 hour. Then, trimethyltinchloride ($Sn(CH_3)_3CI$) (1.10 g, 5.51 mmol)) was added at −78 °C, and the resulting reaction mixture was allowed to warm to room temperature overnight. The reaction mixture was quenched with water, and the product was extracted with hexanes. The organic phase was washed with water, dried over Na_2SO_4, filtered, and evaporated to dryness to give a crude product. The crude product was used for the next step without any further purification. The pure product was obtained as pale orange

oil (2.02 g, 97% yield). ^{1}H NMR ($CDCl_3$): δ 7.54 (d, 1H, *J* = 4.0 Hz), 7.10 (d, 1H, *J* = 4.0 Hz), 2.61 (t, 2H, *J* = 16.0 Hz), 1.22 (s, 20H), 0.88 (t, 3H, *J* = 12.0 Hz), 0.38 (s, 9H) ppm.

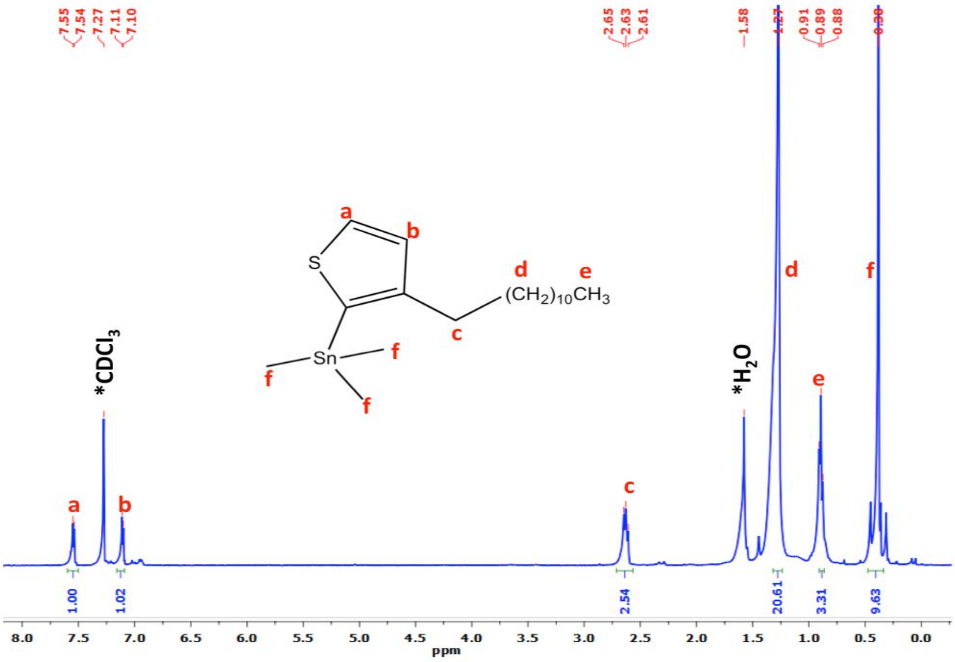

Figure S13. ^{1}H NMR spectrum of **2-trimethyltin-3-dodecylthiophene (2)** in $CDCl_3$ at room temperature. $CDCl_3$ and H_2O peaks in NMR solvent are denoted by asterisks.

Synthesis of 2,8-di-3-dodecylthiophene-indeno[1,2-b]fluorene-6,12-dione (β,β'-C_{12}-TIFDKT): The mixture of 2 (0.726 g, 1.75 mmol), IFDK-Br_2 (0.350 g, 0.795 mmol), and $Pd(PPh_3)_2Cl_2$ (93.0 mg, 0.132 mmol) in anhydrous DMF (55.0 mL) were heated at 125 °C under nitrogen for 48 hours. The reaction mixture was cooled down to RT and evaporated to dryness. Then, the collected product was washed with methanol and filtered to collect the crude product, which was purified by column chromatography on silica gel using $CHCl_3$/hexanes (9:1 (v/v)) as the eluent. Finally, the product was washed with methanol and filtered to afford the final product as a purple solid (0.28 g, 45% yield). m.p.: 152-153 °C. ^{1}H NMR ($CDCl_3$): δ 7.86 (s, 2H), 7.78 (s, 2H), 7.68 (S, 4H), 7.30 (s, 2H), 7.02 (d, 2H, *J* = 4.0 Hz), 2.68 (t, 4H, *J* = 16.0 Hz), 1.24 (s, 40H), 0.86 (t, 6H, *J* = 12.0 Hz) ppm.

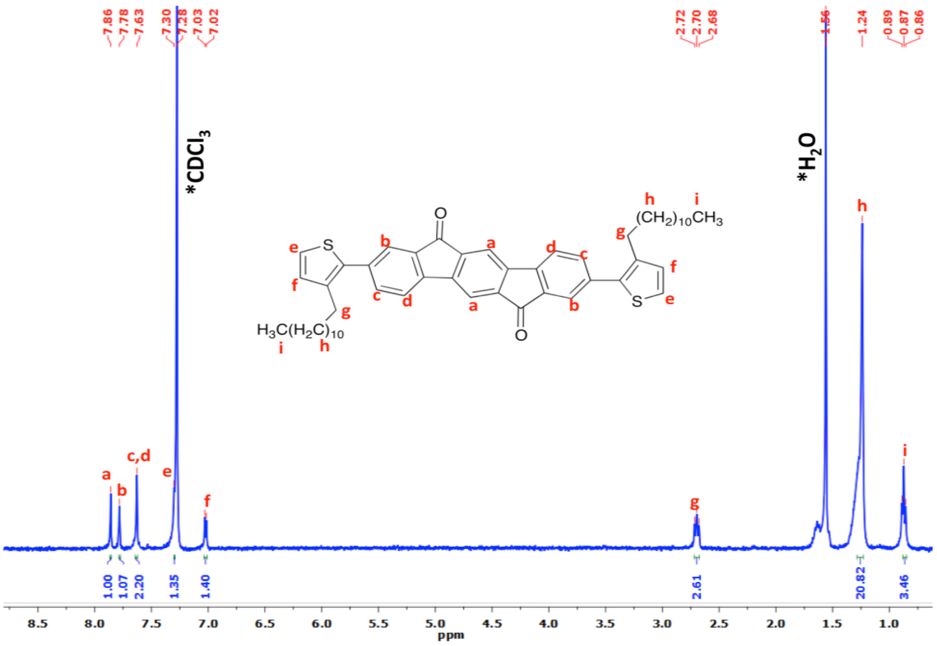

Figure S14. ^{1}H NMR spectrum of **2,8-di-3-dodecylthiophene-indeno[1,2-b]fluorene-6,12-dione (β,β'-C_{12}-TIFDKT)** in $CDCl_3$ at room temperature. $CDCl_3$ and H_2O peaks in NMR solvent are denoted by asterisks.

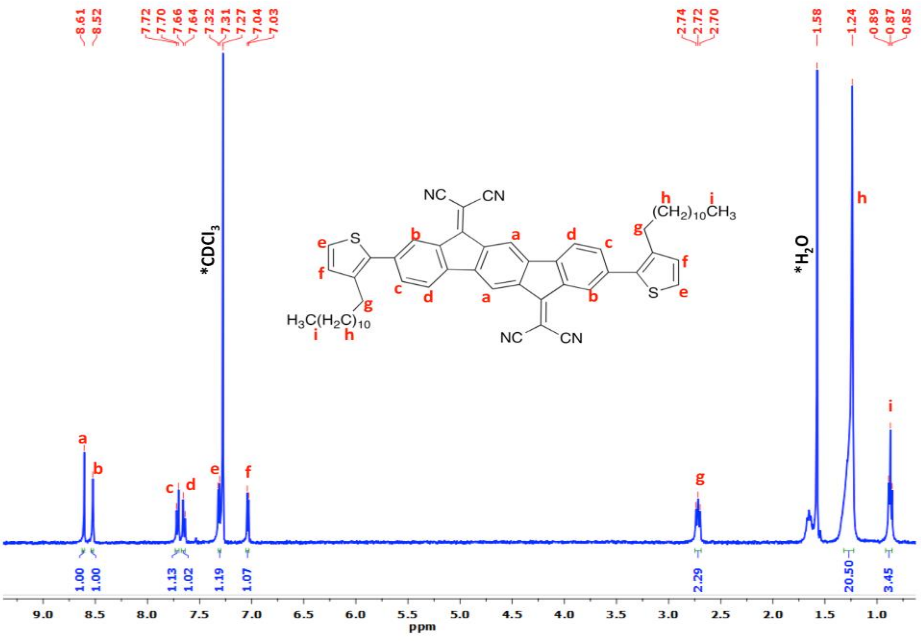

Figure S15. ^{1}H NMR spectrum of **2,8-di-3-dodecylthiophene-indeno[1,2-b]fluorene-6,12-dimalononitrile (β,β'-C_{12}-TIFDMT)** in $CDCl_3$ at room temperature. $CDCl_3$ and H_2O peaks in NMR solvent are denoted by asterisks.

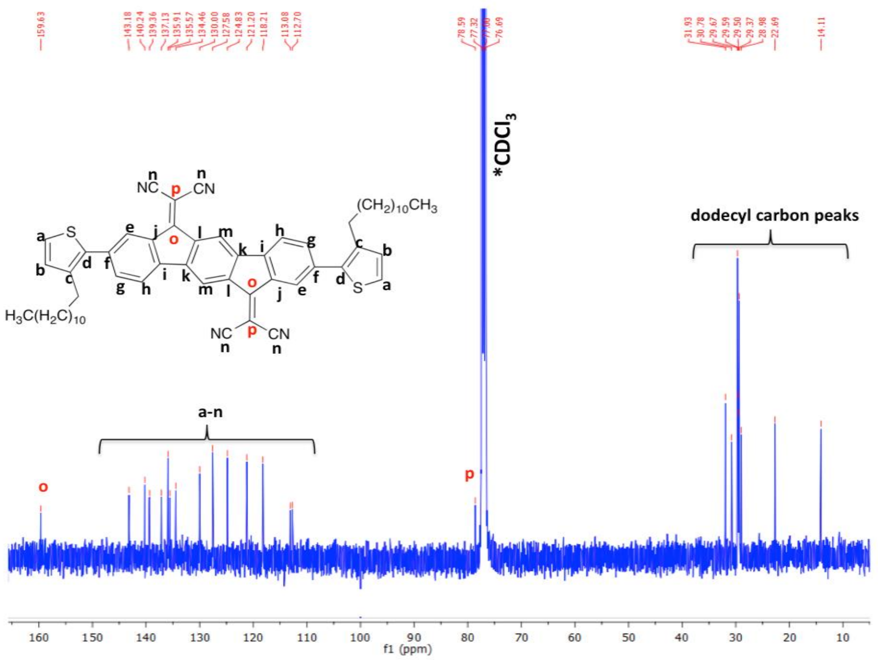

Figure S16. ^{13}C NMR spectrum of **2,8-di-3-dodecylthiophene-indeno[1,2-b]fluorene-6,12-dimalononitrile (β,β'-C_{12}-TIFDMT)** in $CDCl_3$ at room temperature. $CDCl_3$ peak in NMR solvent is denoted by an asterisk.

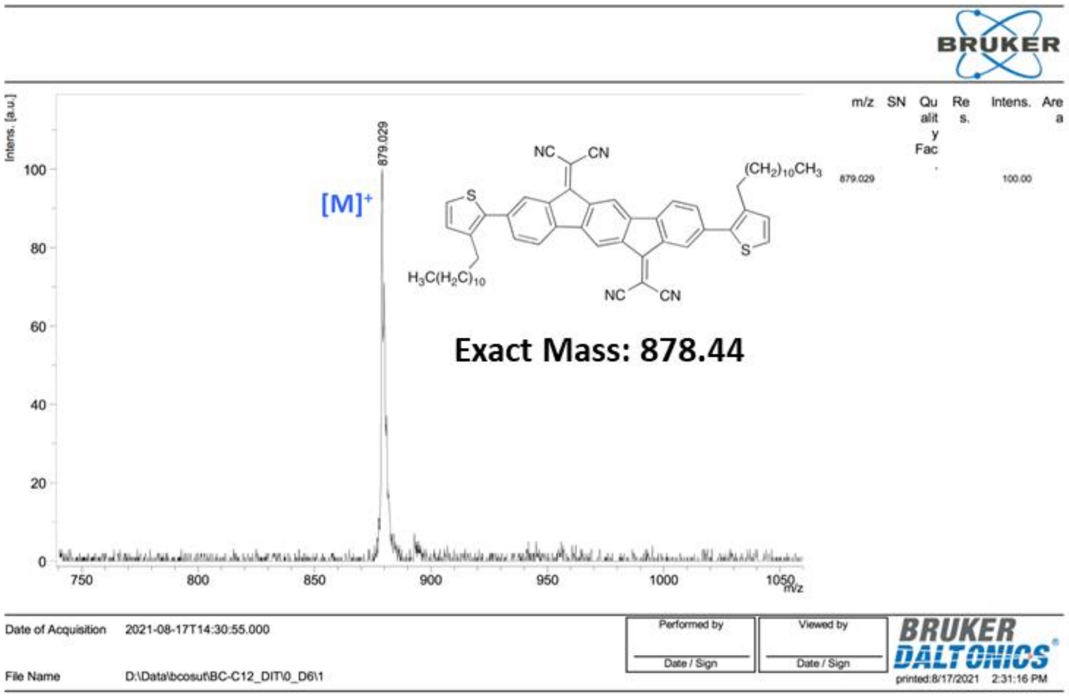

Figure S17. Positive ion and linear mode MALDI TOF-MS spectrum of **2,8-di-3-dodecylthiophene-indeno[1,2-b]fluorene-6,12-dimalononitrile (β,β'-C_{12}-TIFDMT)**.

Synthesis of 3-hexadecylthiophene: To a solution of 1-bromooctane (5.71 mL, 17.00 mmol) in anhydrous THF (30.0 mL) magnesium (0.49 g, 20.08 mmol) and iodine (0.12 g, 0.46 mmol) were added under nitrogen and this mixture was stirred at 85 °C for 3 hours. The resulting Grignard reagent was allowed to cool down to room temperature. Then, the Grignard reagent was added slowly to a solution of 3-bromothiophene (1.45 mL, 15.45 mmol) and $NiCl_2$(dppp) (0.12 g, 0.23 mmol) in anhydrous THF (12.0 mL) at 0 °C under nitrogen. The reaction mixture was allowed to warm to room temperature overnight. The resulting reaction mixture was quenched with water and extracted with dichloromethane. The organic phase was washed with water, dried over Na_2SO_4, filtered, and evaporated to dryness to give a crude product, which was purified by column chromatography on silica gel using hexanes as the eluent. Pure product was afforded as colorless oil (3.04 g, 64% yield). ^{1}H NMR ($CDCl_3$): δ 7.25 (s, 1H), 6.93 (d, 2H, J = 8.0 Hz), 2.61 (t, 2H, J = 16.0 Hz), 1.27 (d, 28H, J = 16.0 Hz), 0.88 (t, 3H, J = 12.0 Hz) ppm.

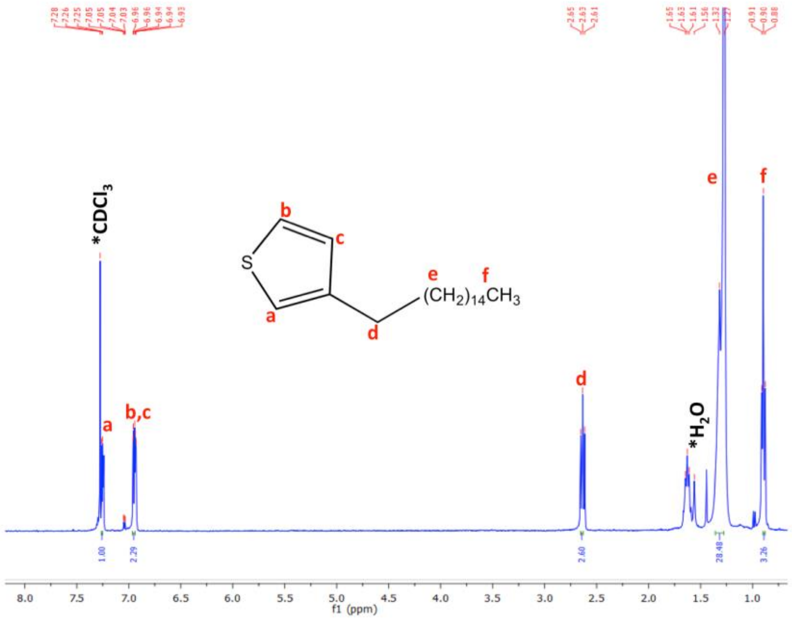

Figure S18. ^{1}H NMR spectrum of **3-hexadecylthiophene** in $CDCl_3$ at room temperature. $CDCl_3$ and H_2O peaks in NMR solvent are denoted by asterisks.

Synthesis of 2-bromo-3-hexadecylthiophene: To a solution of 3-hexadecylthiophene (3.04 g, 9.88 mmol) in chloroform (50 mL): acetic acid (50 mL) (1:1 (v/v)) N-bromosuccinimide (NBS) (1.84 g, 10.38 mmol) was added under nitrogen. The mixture was stirred at room temperature overnight. The resulting reaction mixture was quenched with water and extracted

with dichloromethane. The organic phase was washed with water, dried over Na_2SO_4, filtered, and evaporated to dryness to give a crude product, which was purified by column chromatography on silica gel using hexanes as the eluent. Pure product was afforded as light yellow oil (0.96 g, 25% yield). ^{1}H NMR ($CDCl_3$): δ 7.19 (d, 1H, *J* = 8.0 Hz), 6.80 (d, 1H, *J* = 8.0 Hz), 2.55 (t, 2H, *J* = 16.0 Hz), 1.27 (d, 28H, *J* = 16.0 Hz), 0.88 (t, 3H, *J* = 12.0 Hz) ppm.

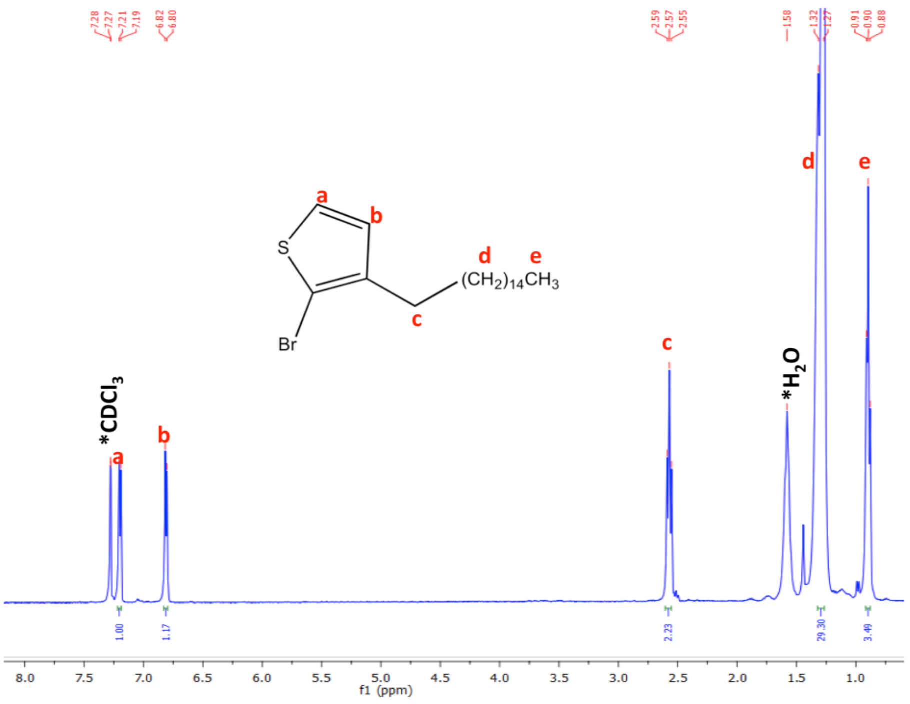

Figure S19. ^{1}H NMR spectrum of **2-bromo-3-hexadecylthiophene** in $CDCl_3$ at room temperature. $CDCl_3$ and H_2O peaks in NMR solvent are denoted by asterisks.

Synthesis of 2-trimethyltin-3-hexadecylthiophene (3): To a solution of 2-bromo-3-hexadecylthiophene (0.96 g, 2.47 mmol) in anhydrous THF (50 ml) at -78 °C n-butyllithium (2.5 M in n-hexane) (1.04 ml, 2.59 mmol) was added dropwise under nitrogen. The mixture was stirred at −78 °C for 1 hour. Then, trimethyltinchloride ($Sn(CH_3)_3CI$) (0.54 g, 2.72 mmol)) was added at −78 °C, and the resulting reaction mixture was allowed to warm to room temperature overnight. The reaction mixture was quenched with water, and the product was extracted with hexanes. The organic phase was washed with water, dried over Na_2SO_4, filtered, and evaporated to dryness to give a crude product. The crude product was used for the next step without any further purification. The pure product was obtained as pale orange oil (1.03 g, 89% yield). ^{1}H NMR ($CDCl_3$): δ 7.54 (d, 1H, *J* = 4.0 Hz), 7.12 (d, 1H, *J* = 4.0 Hz), 2.62 (t, 2H, *J* = 16.0 Hz), 1.28 (d, 28H, *J* = 16.0 Hz), 0.89 (t, 3H, *J* = 12.0 Hz), 0.39 (s, 9H) ppm.

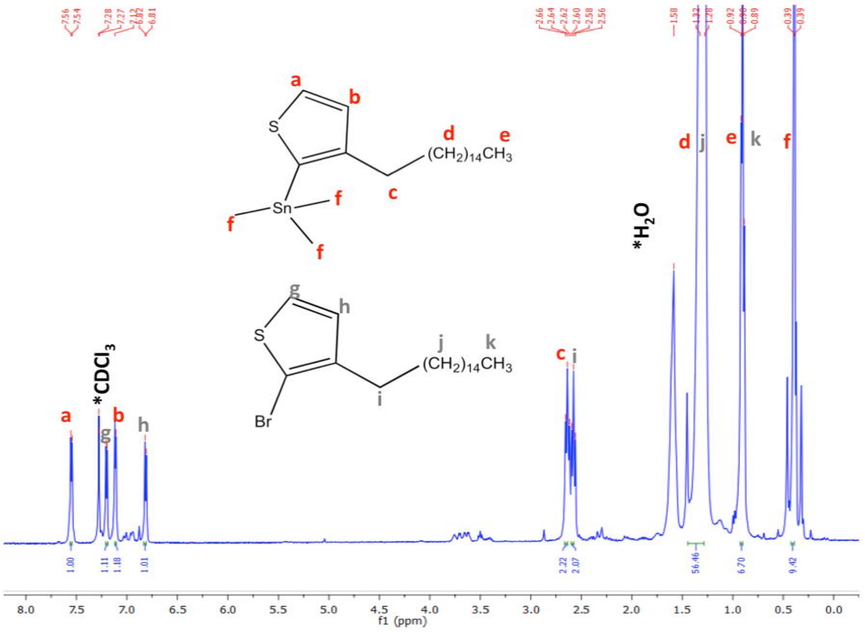

Figure S20. ^{1}H NMR spectrum of **3** in $CDCl_3$ at room temperature. $CDCl_3$ and H_2O peaks in NMR solvent are denoted by asterisks.

Synthesis of 2,8-di-3-hexadecylthiophene-indeno[1,2-b]fluorene-6,12-dione (β,β'-C_{16}-TIFDKT): The mixture of 3 (0.565 g, 1.20 mmol), IFDK-Br_2 (0.240 g, 0.545 mmol), and $Pd(PPh_3)_2Cl_2$ (63.0 mg, 0.090 mmol) in anhydrous DMF (35.0 mL) were heated at 125 °C under nitrogen for 48 hours. The reaction mixture was cooled down to RT and evaporated to dryness. Then, the collected product was washed with methanol and filtered to collect the crude product, which was purified by column chromatography on silica gel using $CHCl_3$/hexanes (8:2 (v/v)) as the eluent. Finally, the product was washed with methanol and filtered to afford the final product as a purple solid (39.5 mg, 9% yield). m.p.: 144-145 °C. ^1H NMR ($CDCl_3$): δ 7.86 (s, 2H), 7.78 (s, 2H), 7.63 (S, 4H), 7.30 (d, 2H, J = 4.0 Hz), 7.02 (d, 2H, J = 4.0 Hz) 2.69 (t, 4H, J = 16.0 Hz), 1.24 (s, 56H), 0.86 (t, 6H, J = 16.0 Hz) ppm.

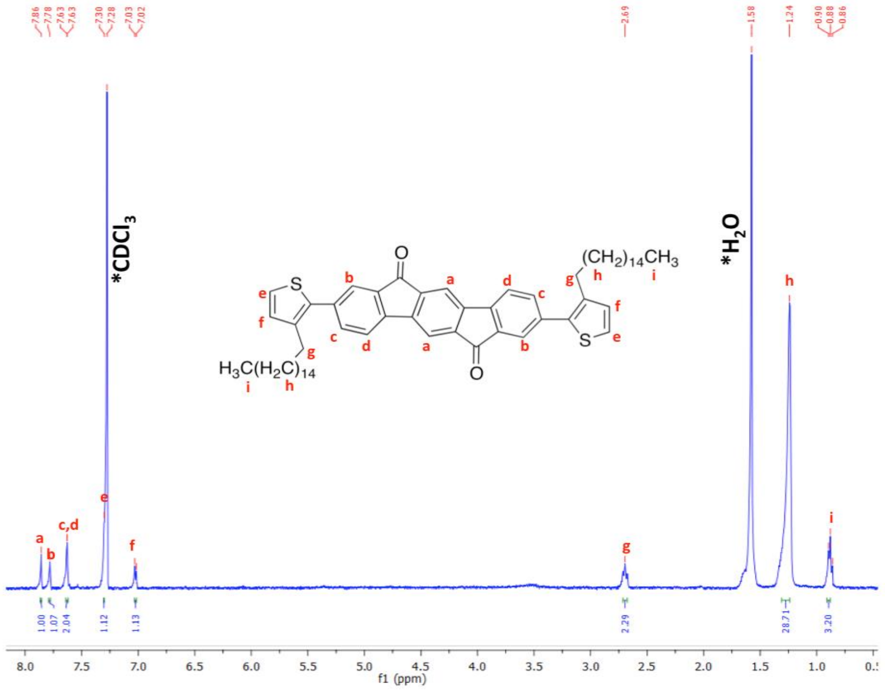

Figure S21. ^{1}H NMR spectrum of **2,8-di-3-hexadecylthiophene-indeno[1,2-b]fluorene-6,12-dione (β,β'-C$_{16}$-TIFDKT)** in $CDCl_3$ at room temperature. $CDCl_3$ and H_2O peaks in NMR solvent are denoted by asterisks.

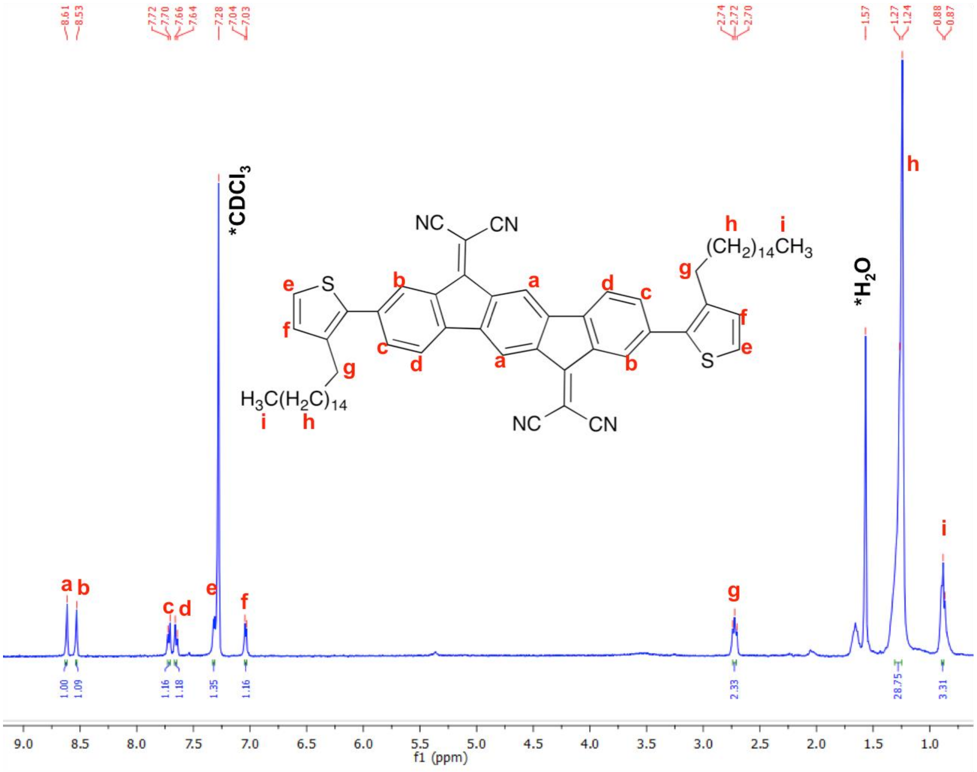

Figure S22. ^{1}H NMR spectrum of **2,8-di-3-hexadecylthiophene-indeno[1,2-b]fluorene-6,12-dimalononitrile (β,β'-C$_{16}$-TIFDMT)** in $CDCl_3$ at room temperature. $CDCl_3$ and H_2O peaks in NMR solvent are denoted by asterisks.

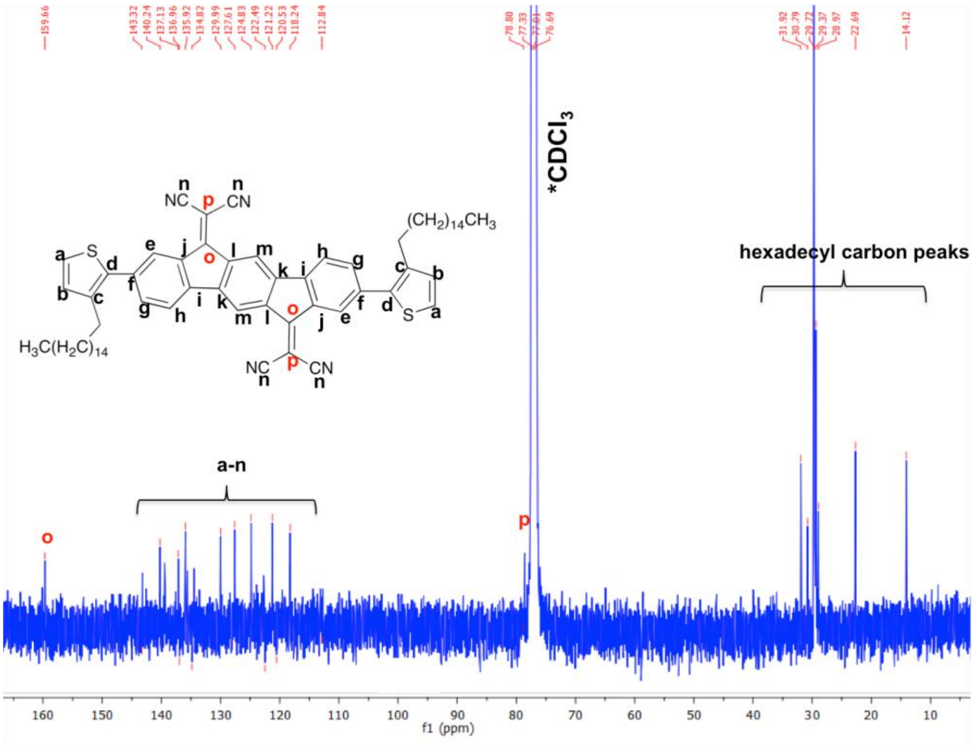

Figure S23. ^{13}C NMR spectrum of **2,8-di-3-hexadecylthiophene-indeno[1,2-b]fluorene-6,12-dimalononitrile (β,β'-C_{16}-TIFDMT)** in $CDCl_3$ at room temperature. $CDCl_3$ peak in NMR solvent is denoted by an asterisk.

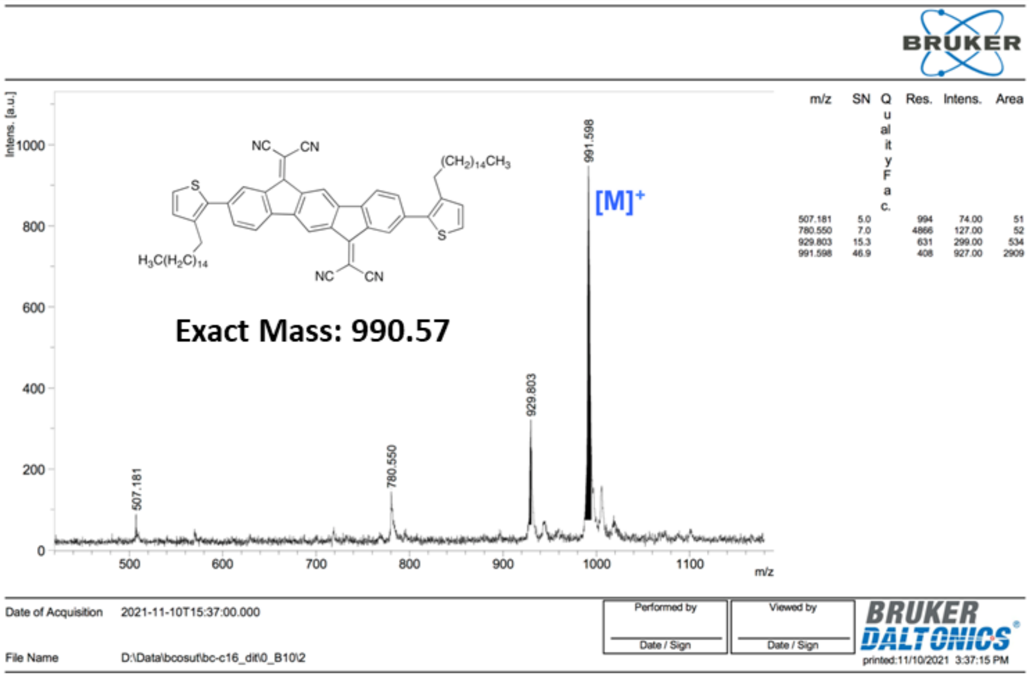

Figure S24. Positive ion and linear mode MALDI TOF-MS spectrum of **2,8-di-3-hexadecylthiophene-indeno[1,2-b]fluorene-6,12-dimalononitrile (β,β'-C_{16}-TIFDMT)**.

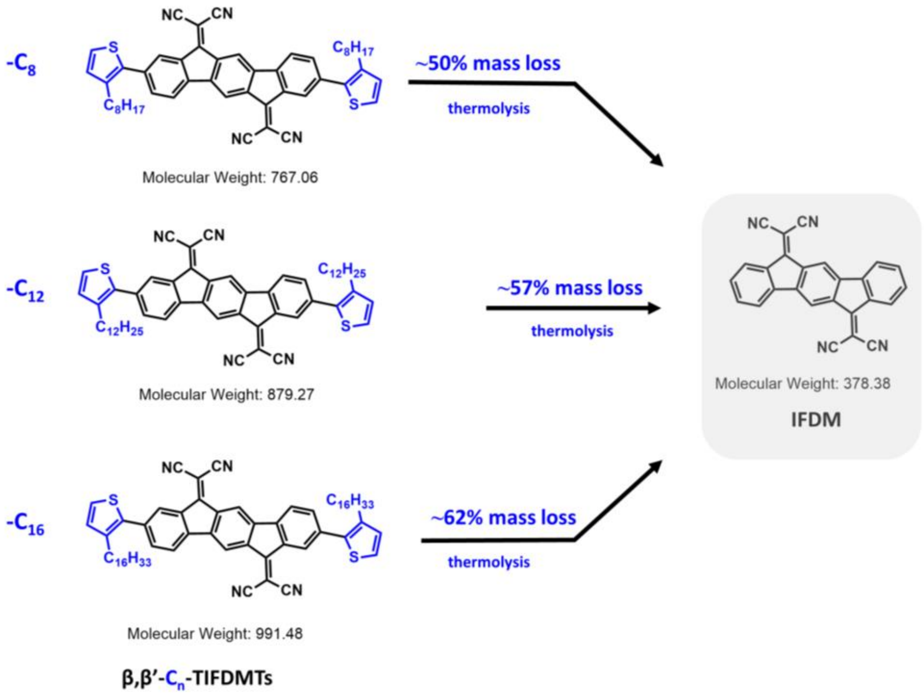

Figure S25. The final mass loss percentages and the proposed thermolysis reactions for the **β,β'-C8-TIFDMT**, **β,β'-C12-TIFDMT**, and **β,β'-C16-TIFDMT** solids during thermogravimetric analyses conducted at a temperature ramp of 10 °C min^{-1} under N_2.

Measurement and calculation of thermodynamic quantities. Differential scanning calorimetery (DSC) measurements were performed on Mettler Toledo DSC822e at a heating temperature rate of 10 °C min^{-1} under N_2. The obtained DSC curves for the **β,β'-C_8-TIFDMT**, **β,β'-C_{12}-TIFDMT**, and **β,β'-C_{16}-TIFDMT** solids are shown in Figure 2(b). The phase transition temperatures (T_{trans}) and the corresponding enthalpies (ΔH_{trans}) upon heating are summarized in Table S2 below. All phase transition entropies (ΔS_{trans})[20] are calculated using the values of ΔH_{trans} and T_{trans} based on the equation $\Delta S_{trans} = \Delta H_{trans}/T_{trans}$. The total enthalpy ($\Delta H_{S\rightarrow I}$) and entropy ($\Delta S_{S\rightarrow I}$) values for the solid-to-isotropic liquid transition are calculated by the summation of all phase transitions.

Table S2. Thermodynamic quantities associated with phase transitions of the **β,β'-C_8-TIFDMT**, **β,β'-C_{12}-TIFDMT**, and **β,β'-C_{16}-TIFDMT** solids observed upon heating during DSC measurements. Melting point (T_{mp}) was measured via conventional melting temperature measurements.

**Molecule**	**ΔH_{trans} (kJ/mol)**	**T_{mp} (°C)**	**T_{trans} (°C)**	**ΔS_{trans} (J/K·mol)**
**β,β'-C_8-TIFDMT**	44.07<br>$\Delta H_{S\rightarrow I}$ = 44.07	237-238	239.8	85.91<br>$\Delta S_{S\rightarrow I}$ = 85.91
**β,β'-C_{12}-TIFDMT**	45.34<br>16.86<br>25.53<br>$\Delta H_{S\rightarrow I}$ = 87.73	231-232	231.0<br>175.1<br>86.8	89.93<br>37.61<br>70.93<br>$\Delta S_{S\rightarrow I}$ = 198.47
**β,β'-C_{16}-TIFDMT**	61.90<br>22.25<br>34.26<br>$\Delta H_{S\rightarrow I}$ = 118.41	221-222	222.3<br>165.9<br>77.6	124.94<br>50.68<br>97.68<br>$\Delta S_{S\rightarrow I}$ = 273.3

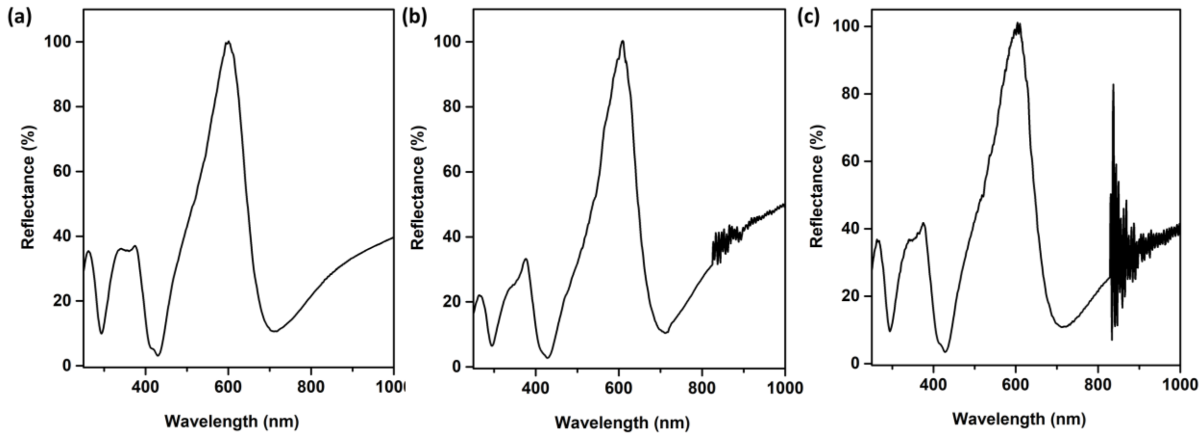

Figure S26. The UV-vis diffuse reflectance spectra of **β,β'-C_8-TIFDMT** (a), **β,β'-C_{12}-TIFDMT** (b), and **β,β'-C_{16}-TIFDMT** (c) as spin-coated thin-films on p^{++}-Si/SiO_2/PS-brush (M_n = 5 kDa) (annealed at 150 °C) collected using an integrating sphere.

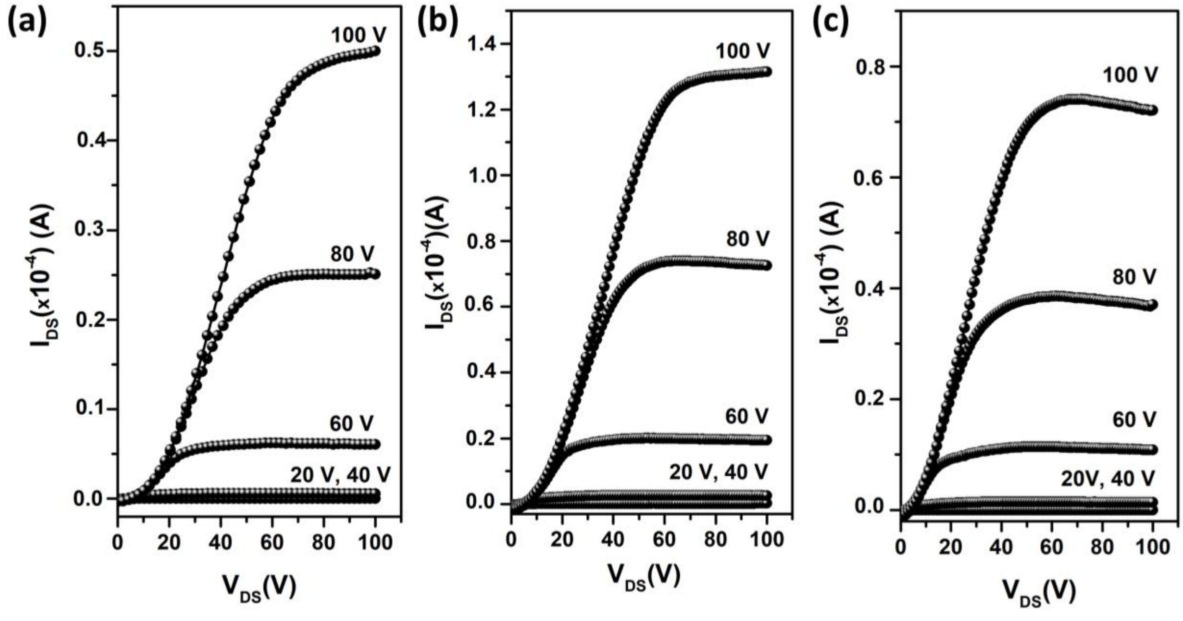

Figure S27. Output plots (V_G = 0-100 V) measured in ambient for p^{++}-Si/SiO_2/PS-brush (M_n= 5 kDa)/semiconductor/Au OFET devices fabricated based on the semiconductor molecules **β,β'-C_8-TIFDMT** (a), **β,β'-C_{12}-TIFDMT** (b), and **β,β'-C_{16}-TIFDMT** (c). The annealing temperature for each device is 150 °C.

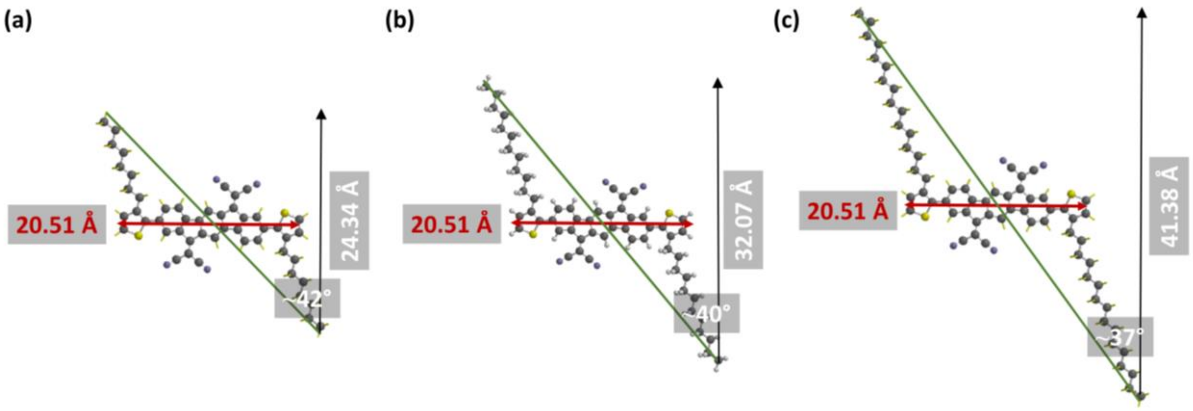

Figure S28. DFT-Computed molecular dimensions for (a) **β,β'-C$_8$-TIFDMT**, (b) **β,β'-C$_{12}$-TIFDMT**, and (c) **β,β'-C$_{16}$-TIFDMT**.

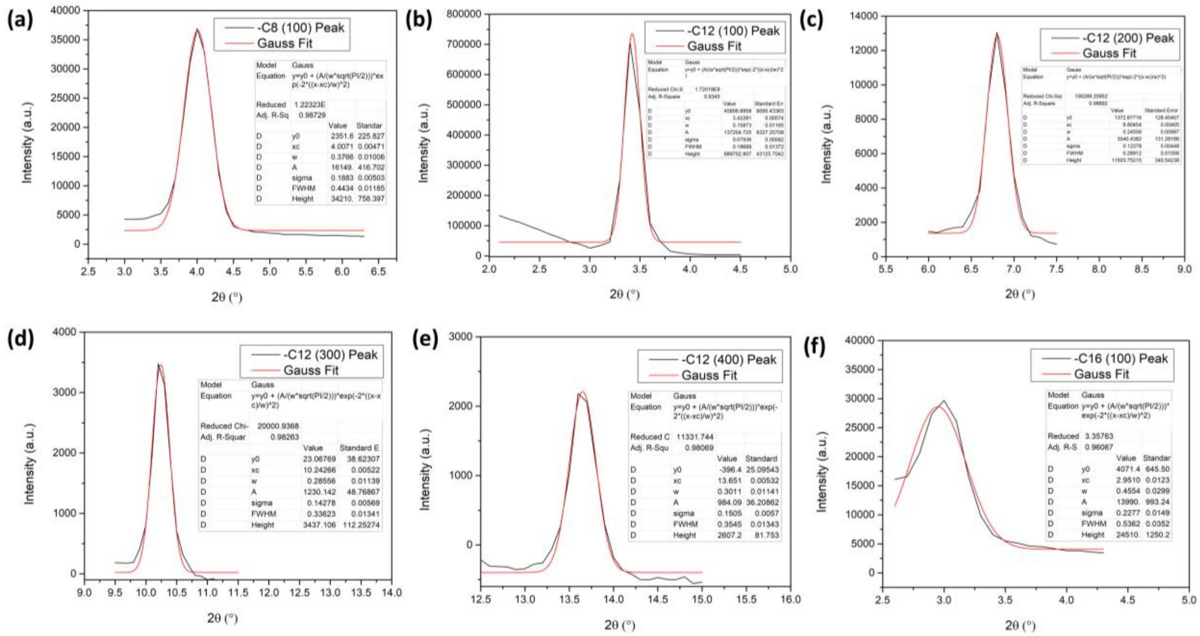

Figure S29. The Gauss fittings for the (a) **β,β'-C$_8$-TIFDMT**'s (100) GIXRD peak, (b), (c), (d), (e) **β,β'-C$_{12}$-TIFDMT**'s (100), (200), (300), and (400) GIXRD peaks, and (f) **β,β'-C$_{16}$-TIFDMT**'s (100) GIXRD peak, and the corresponding Adj. R-squared, β (fwhm), and 2θ (peak position) values to be used in the Scherrer Equation.

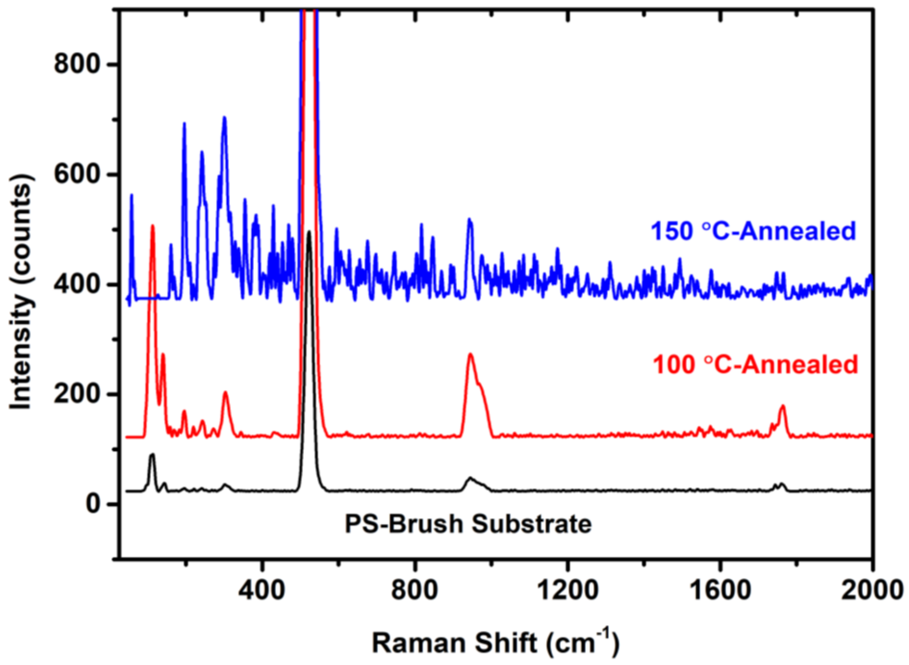

Figure S30. Raman spectra of p^{++}-Si/SiO$_2$/PS-brush (M_n = 5 kDa) substrates (black line), and **β,β'-C$_{12}$-TIFDMT** thin-film on p^{++}-Si/SiO$_2$/PS-brush (M_n = 5 kDa) after 100 °C (red line) and 150 °C thermal annealing (blue line) (for 30 min) under vacuum.

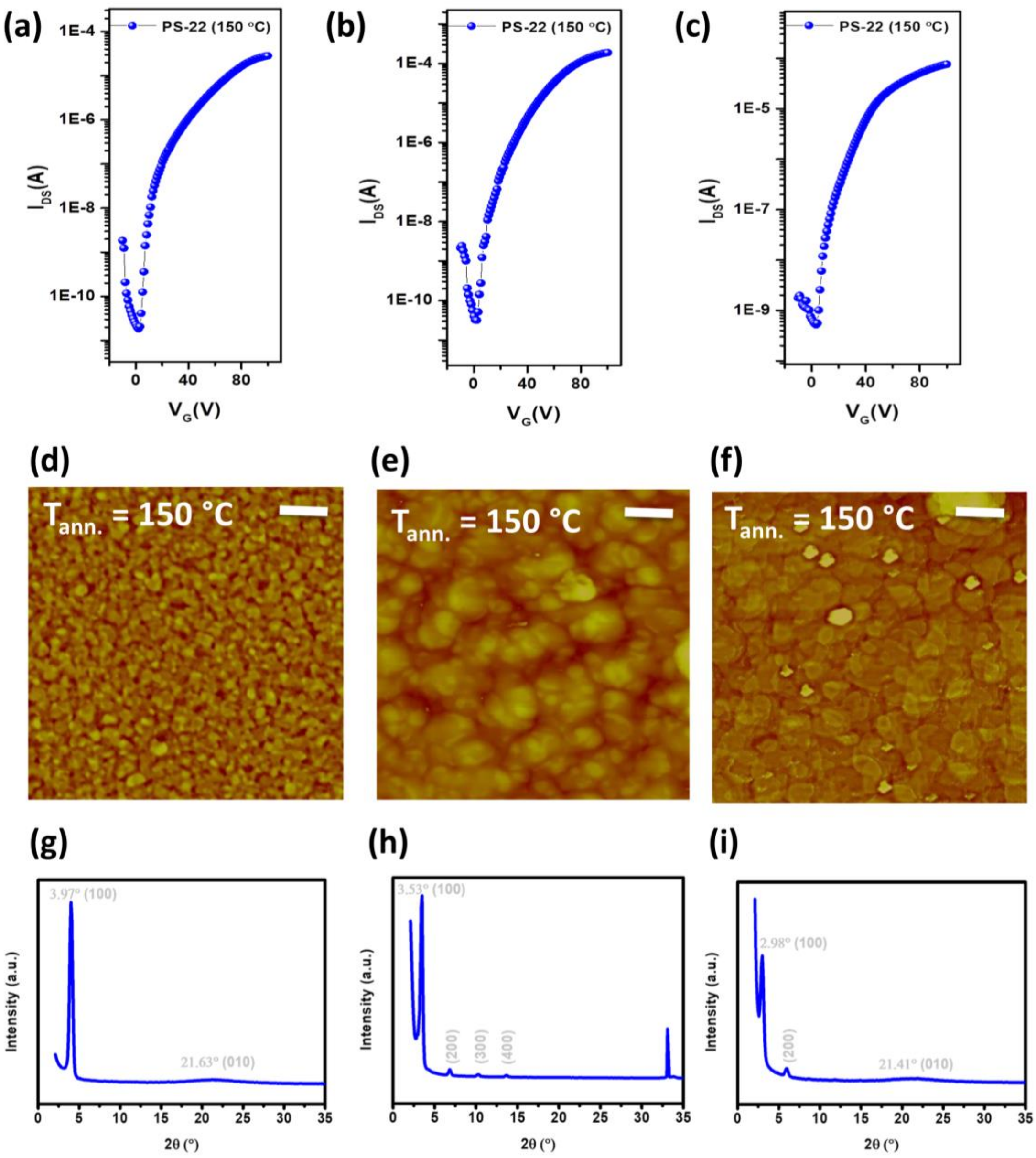

Figure S31. Transfer plots (V_{DS} = 100 V) measured in ambient for p^{++}-Si/SiO_2/PS-brush (M_n = 22 kDa)/semiconductor/Au OFET devices fabricated based on the semiconductor molecules **β,β'-C_8-TIFDMT** (a), **β,β'-C_{12}-TIFDMT** (b), and **β,β'-C_{16}-TIFDMT** (c). The annealing temperature for each device is 150 °C. Top-view atomic force microscopy (AFM) topography images for spin-coated **β,β'-C_8-TIFDMT** (d), **β,β'-C_{12}-TIFDMT** (e), and **β,β'-C_{16}-TIFDMT** (f) thin-films on p^{++}-Si/SiO_2/PS-brush (M_n = 22 kDa) after thermal annealing at temperature of 150 °C. Scales bars denote 2 μm. The GIXRD scans of **β,β'-C_8-TIFDMT** (g), **β,β'-C_{12}-TIFDMT** (h), and **β,β'-C_{16}-TIFDMT** (i) thin-films on p^{++}-Si/SiO_2/PS-brush (M_n = 22kDa) after thermal annealing at temperature of 150 °C with the 2θ diffraction peaks and the assigned out-of-plane crystallographic planes ((100), (200), (300), (400), and (010)).

REFERENCES

(1) Qiao, Y.; Guo, Y.; Yu, C.; Zhang, F.; Xu, W.; Liu, Y.; Zhu, D. Diketopyrrolopyrrole-Containing Quinoidal Small Molecules for High-Performance, Air-Stable, and Solution-Processable n-Channel Organic Field-Effect Transistors. *J. Am. Chem. Soc.* **2012**, *134* (9), 4084–4087. https://doi.org/10.1021/ja3003183.

(2) Jung, B. J.; Lee, K.; Sun, J.; Andreou, A. G.; Katz, H. E. Air-Operable, High-Mobility Organic Transistors with Semifluorinated Side Chains and Unsubstituted Naphthalenetetracarboxylic Diimide Cores: High Mobility and Environmental and Bias Stress Stability from the Perfluorooctylpropyl Side Chain. *Adv. Funct. Mater.* **2010**, *20* (17), 2930–2944. https://doi.org/10.1002/adfm.201000655.

(3) See, K. C.; Landis, C.; Sarjeant, A.; Katz, H. E. Easily Synthesized Naphthalene Tetracarboxylic Diimide Semiconductors with High Electron Mobility in Air. *Chem. Mater.* **2008**, *20* (11), 3609–3616. https://doi.org/10.1021/cm7032614.

(4) Schmidt, R.; Oh, J. H.; Sun, Y. Sen; Deppisch, M.; Krause, A. M.; Radacki, K.; Braunschweig, H.; Könemann, M.; Erk, P.; Bao, Z.; Würthner, F. High-Performance Air-Stable n-Channel Organic Thin Film Transistors Based on Halogenated Perylene Bisimide Semiconductors. *J. Am. Chem. Soc.* **2009**, *131* (17), 6215–6228. https://doi.org/10.1021/ja901077a.

(5) Gsänger, M.; Oh, J. H.; Könemann, M.; Höffken, H. W.; Krause, A. M.; Bao, Z.; Würthner, F. A Crystal-Engineered Hydrogen-Bonded Octachloroperylene Diimide with a Twisted Core: An n-Channel Organic Semiconductor. *Angew. Chemie - Int. Ed.* **2010**, *49* (4), 740–743. https://doi.org/10.1002/anie.200904215.

(6) Hak, J.; Sabin-Lucian, S.; Lee, W. Y.; Könemann, M.; Höffken, H. W.; Röger, C.; Schmidt, R.; Chung, Y.; Chen, W. C.; Würthner, F.; Bao, Z. High-Performance Air-Stable n-Type Organic Transistors Based on Core-Chlorinated Naphthalene Tetracarboxylic Diimides. *Adv. Funct. Mater.* **2010**, *20* (13), 2148–2156. https://doi.org/10.1002/adfm.201000425.

(7) Zhang, F.; Hu, Y.; Schuettfort, T.; Di, C. A.; Gao, X.; McNeill, C. R.; Thomsen, L.; Mannsfeld, S. C. B.; Yuan, W.; Sirringhaus, H.; Zhu, D. Critical Role of Alkyl Chain Branching of Organic Semiconductors in Enabling Solution-Processed N-Channel Organic Thin-Film Transistors with Mobility of up to 3.50 Cm2 V-1 s-1. *J. Am. Chem. Soc.* **2013**, *135* (6), 2338–2349. https://doi.org/10.1021/ja311469y.

(8) Hu, Y.; Qin, Y.; Gao, X.; Zhang, F.; Di, C. A.; Zhao, Z.; Li, H.; Zhu, D. One-Pot Synthesis of Core-Expanded Naphthalene Diimides: Enabling N-Substituent Modulation for Diverse n-Type Organic Materials. *Org. Lett.* **2012**, *14* (1), 292–295. https://doi.org/10.1021/ol203059r.

(9) Zhang, J.; Tan, L.; Jiang, W.; Hu, W.; Wang, Z. N-Alkyl Substituted Di(Perylene Bisimides) as Air-Stable Electron Transport Materials for Solution-Processible Thin-Film Transistors with Enhanced Performance. *J. Mater. Chem. C* **2013**, *1* (19), 3200–3206. https://doi.org/10.1039/c3tc30156g.

(10) Zhang, D.; Zhao, L.; Zhu, Y.; Li, A.; He, C.; Yu, H.; He, Y.; Yan, C.; Goto, O.; Meng, H. Effects of P-(Trifluoromethoxy)Benzyl and p-(Trifluoromethoxy)Phenyl Molecular Architecture on the Performance of Naphthalene Tetracarboxylic Diimide-Based Air-Stable n-Type Semiconductors. *ACS Appl. Mater. Interfaces* **2016**, *8* (28), 18277–18283. https://doi.org/10.1021/acsami.6b04753.

(11) Gao, X.; Di, C. A.; Hu, Y.; Yang, X.; Fan, H.; Zhang, F.; Liu, Y.; Li, H.; Zhu, D. Core-Expanded Naphthalene Diimides Fused with 2-(1,3-Dithiol-2-Ylidene) Malonitrile Groups for High-Performance, Ambient-Stable, Solution-Processed n-Channel Organic Thin Film Transistors. *J. Am. Chem. Soc.* **2010**, *132* (11), 3697–3699.

https://doi.org/10.1021/ja910667y.
(12) Zhang, C.; Zang, Y.; Gann, E.; McNeill, C. R.; Zhu, X.; Di, C. A.; Zhu, D. Two-Dimensional π-Expanded Quinoidal Terthiophenes Terminated with Dicyanomethylenes as n-Type Semiconductors for High-Performance Organic Thin-Film Transistors. *J. Am. Chem. Soc.* **2014**, *136* (46), 16176–16184. https://doi.org/10.1021/ja510003y.
(13) Zhang, C.; Zang, Y.; Zhang, F.; Diao, Y.; McNeill, C. R.; Di, C. an; Zhu, X.; Zhu, D. Pursuing High-Mobility n-Type Organic Semiconductors by Combination of “Molecule-Framework” and “Side-Chain” Engineering. *Adv. Mater.* **2016**, *28* (38), 8456–8462. https://doi.org/10.1002/adma.201602598.
(14) Wu, Q.; Li, R.; Hong, W.; Li, H.; Gao, X.; Zhu, D. Dicyanomethylene-Substituted Fused Tetrathienoquinoid for High-Performance, Ambient-Stable, Solution-Processable n-Channel Organic Thin-Film Transistors. *Chem. Mater.* **2011**, *23* (13), 3138–3140. https://doi.org/10.1021/cm201326c.
(15) Velusamy, A.; Yu, C.; Afraj, S. N.; Lin, C.; Lo, W.; Yeh, C.; Wu, Y.; Hsieh, H.; Chen, J.; Lee, G.; Tung, S.; Liu, C.; Chen, M.; Facchetti, A. Thienoisoindigo (TII)-Based Quinoidal Small Molecules for High-Performance N-Type Organic Field Effect Transistors. *Adv. Sci.* **2021**, *8* (1), 2002930. https://doi.org/10.1002/advs.202002930.
(16) Ozdemir, R.; Park, S.; Deneme, İ.; Park, Y.; Zorlu, Y.; Alidagi, H. A.; Harmandar, K.; Kim, C.; Usta, H. Triisopropylsilylethynyl-Substituted Indenofluorenes: Carbonyl versus Dicyanovinylene Functionalization in One-Dimensional Molecular Crystals and Solution-Processed n-Channel OFETs. *Org. Chem. Front.* **2018**, *5* (20), 2912–2924. https://doi.org/10.1039/C8QO00856F.
(17) Ozdemir, R.; Choi, D.; Ozdemir, M.; Kim, H.; Kostakoğlu, S. T.; Erkartal, M.; Kim, H.; Kim, C.; Usta, H. A Solution-Processable Liquid-Crystalline Semiconductor for Low-Temperature-Annealed Air-Stable N-Channel Field-Effect Transistors. *ChemPhysChem* **2017**, *18* (7), 850–861. https://doi.org/10.1002/cphc.201601430.
(18) Ozdemir, R.; Choi, D.; Ozdemir, M.; Kwon, G.; Kim, H.; Sen, U.; Kim, C.; Usta, H. Ultralow Bandgap Molecular Semiconductors for Ambient-Stable and Solution-Processable Ambipolar Organic Field-Effect Transistors and Inverters. *J. Mater. Chem. C* **2017**, *5* (9), 2368–2379. https://doi.org/10.1039/C6TC05079D.
(19) Usta, H.; Risko, C.; Wang, Z.; Huang, H.; Deliomeroglu, M. K.; Zhukhovitskiy, A.; Facchetti, A.; Marks, T. J. Design, Synthesis, and Characterization of Ladder-Type Molecules and Polymers. Air-Stable, Solution-Processable n -Channel and Ambipolar Semiconductors for Thin-Film Transistors via Experiment and Theory. *J. Am. Chem. Soc.* **2009**, *131* (15), 5586–5608. https://doi.org/10.1021/ja809555c.
(20) Inoue, S.; Shinamura, S.; Sadamitsu, Y.; Arai, S.; Horiuchi, S.; Yoneya, M.; Takimiya, K.; Hasegawa, T. Extended and Modulated Thienothiophenes for Thermally Durable and Solution-Processable Organic Semiconductors. *Chem. Mater.* **2018**, *30* (15), 5050–5060. https://doi.org/10.1021/acs.chemmater.8b01339.
